# Supplementary material for: Biomimetic KcsA channels enabled by 1D MOF-in-2D COF
Source: Nat Commun. 2025 Oct 14;16:9099. doi: 10.1038/s41467-025-63265-w (PMC12521740; doi:10.1038/s41467-025-63265-w)
Supplement: Supplementary file 1 — Supplementary Information [file 41467_2025_63265_MOESM1_ESM.pdf]

Supplementary Information

Biomimetic KcsA channels enabled by 1D MOF-in-2D COF

Qian Sun[1], Pengjia Dou[1], Jingcheng Du[1], Ayan Yao[1], Dong Cao[1], Ji Ma[1], Shabi Ul Hassan[1], Jian Guan[1], Jiangtao Liu[1, *]

[1]Department of Environmental Science and Engineering, University of Science and Technology of China, 230026, China

*Corresponding author

Email: jiangtaoliu@ustc.edu.cn

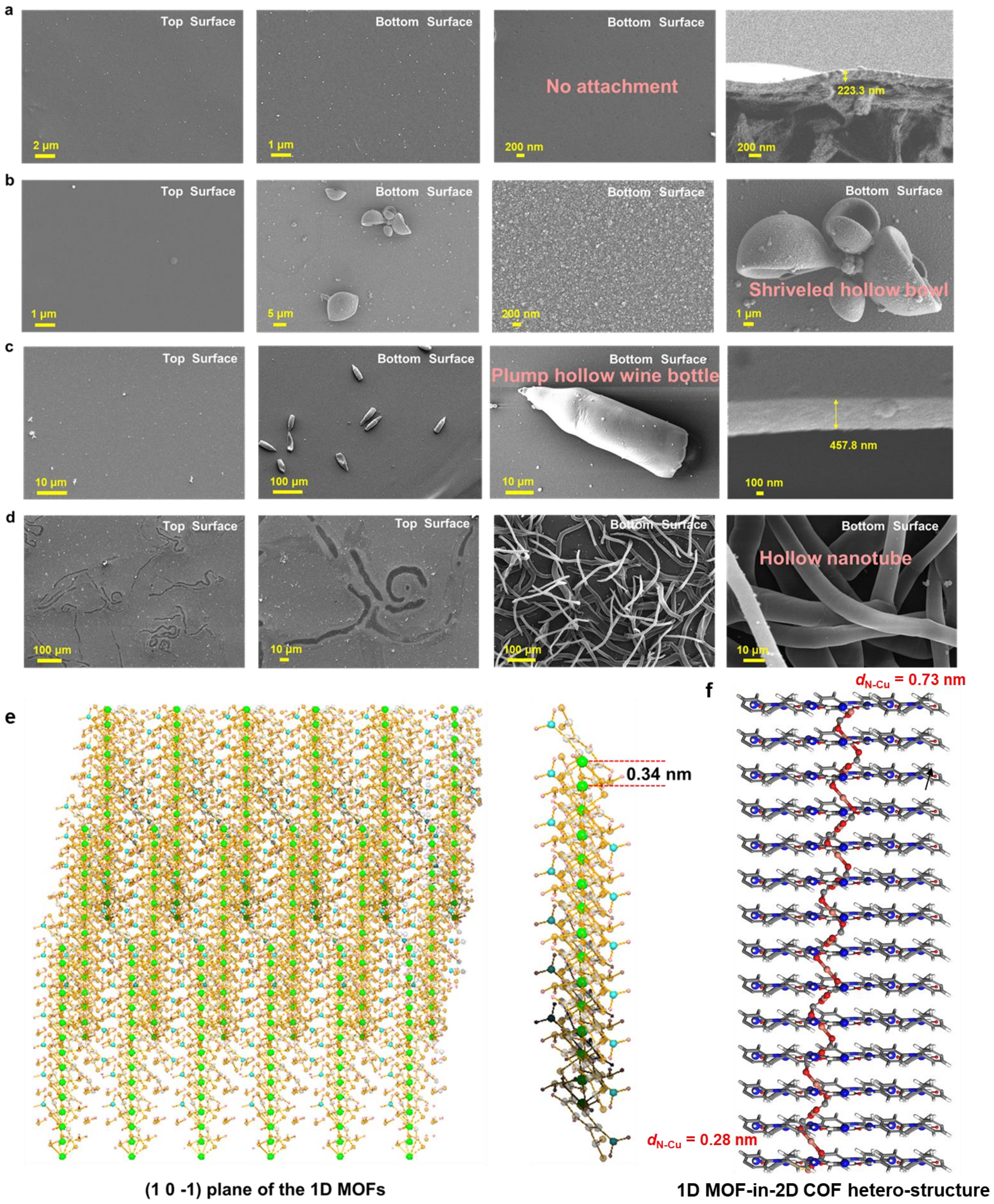

Supplementary Figure 1. SEM images of the pristine COF membrane: (a) Monomer concentration: 0.5 mM TAPA + 0.5 mM TFP. (b) Monomer concentration: 1 mM TAPA + 1 mM TFP. (c) Monomer concentration: 2 mM TAPA + 2 mM TFP. (d)

Monomer concentration: 4 mM TAPA + 4 mM TFP. (e) The (1 0 -1) plane of the 1D MOFs. (f) The simulated 1D MOF-in-2D COF hetero-structure (Some atoms are omitted for clarity.).

Supplementary Notes: The distance between Cu center from MOFs and its neighboring N atom from COFs ranges from 0.28 to 0.73 nm, which confirms the short-range coordination interaction between MOFs and COFs. It's worth mentioning that the surface morphology of pristine COF membrane can be easily tuned by changing monomer concentrations. With the hierarchical increase of monomer concentrations, shriveled hollow bowl, plump hollow wine bottle and hollow nanotube shaped COFs appear on the bottom surfaces. When the monomer concentration is low, the top and bottom surfaces of the COF membrane are almost as smooth, and no attachments are observed on the bottom surface (monomer concentration: 0.25 mM TAPA + 0.25 mM TFP). With the hierarchical increase of monomer concentrations, shriveled hollow bowl (monomer concentration: 1 mM TAPA + 1 mM TFP) and plump hollow wine bottle (monomer concentration: 2 mM TAPA + 2 mM TFP) shaped COFs appear on the bottom surfaces but the top surfaces are still smooth. When the monomer concentration is increased again (monomer concentration: 4 mM TAPA + 4 mM TFP), its bottom surface is covered with a large number of hollow nanotube-shaped COFs, and the indentation caused by nanotubes appears on the top surface.

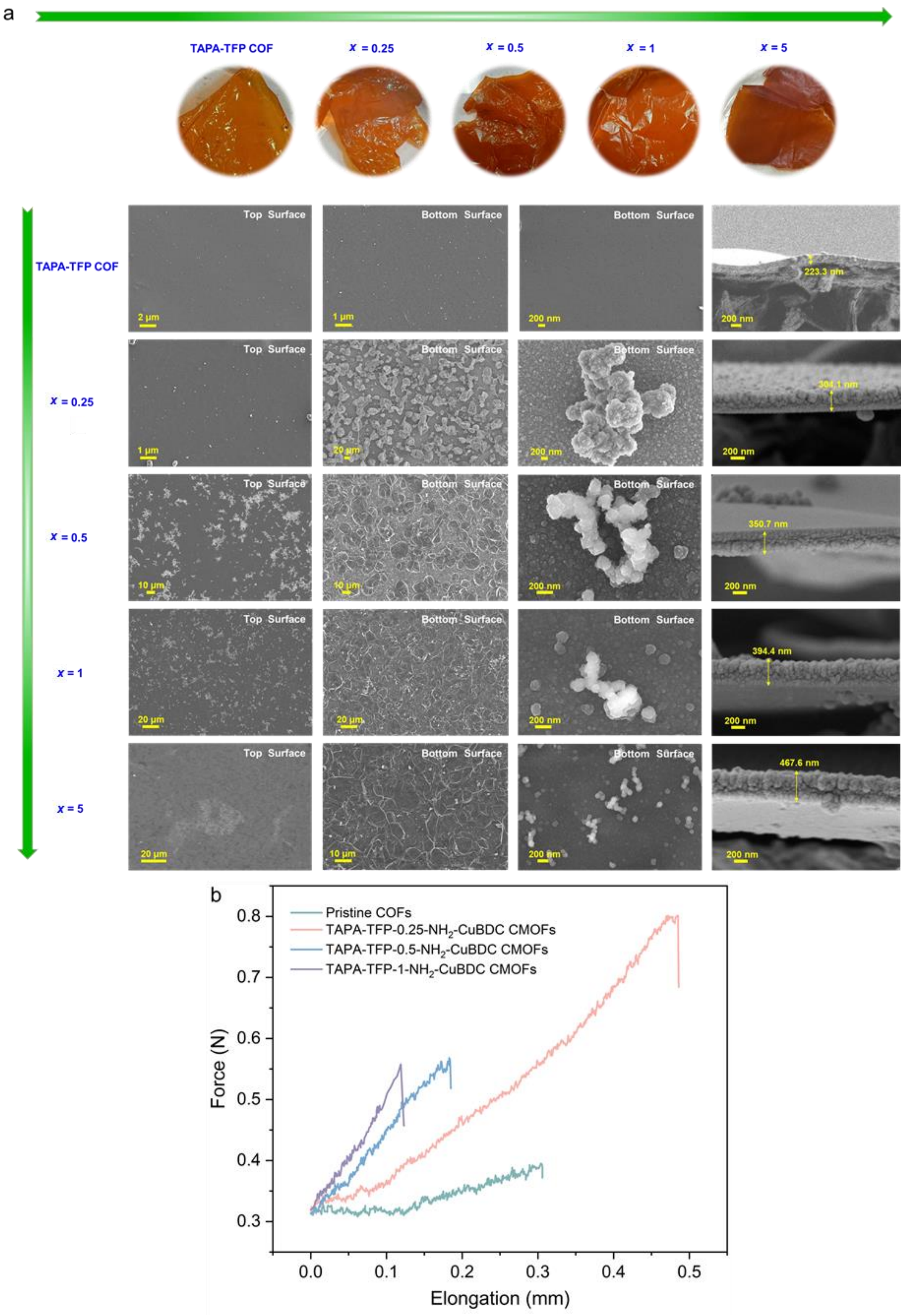

Supplementary Figure 2. (a) Optical photographs and SEM images of prepared TAPA-TFP-*x*-NH_2-CuBDC CMOF composite membranes (monomer concentration: 0.5 mM TAPA + 0.5 mM

TFP). (b) Force-elongation curves tested using a microcomputer-controlled electronic tensile testing machine.

Supplementary Notes: The pristine COF membrane shows a thickness of 223.3 nm, while the TAPA-TFP-0.25-NH_2-CuBDC, TAPA-TFP-0.5-NH_2-CuBDC, TAPA-TFP-1-NH_2-CuBDC and TAPA-TFP-5-NH_2-CuBDC CMOF membranes respectively exhibit the thicknesses of 304.1 nm, 350.7 nm, 394.4 nm and 467.6 nm. CMOF membranes demonstrate superior mechanical properties compared to pristine COF membranes. Furthermore, as the MOF ligand concentration increases, the mechanical properties of CMOF membranes tends to decline.

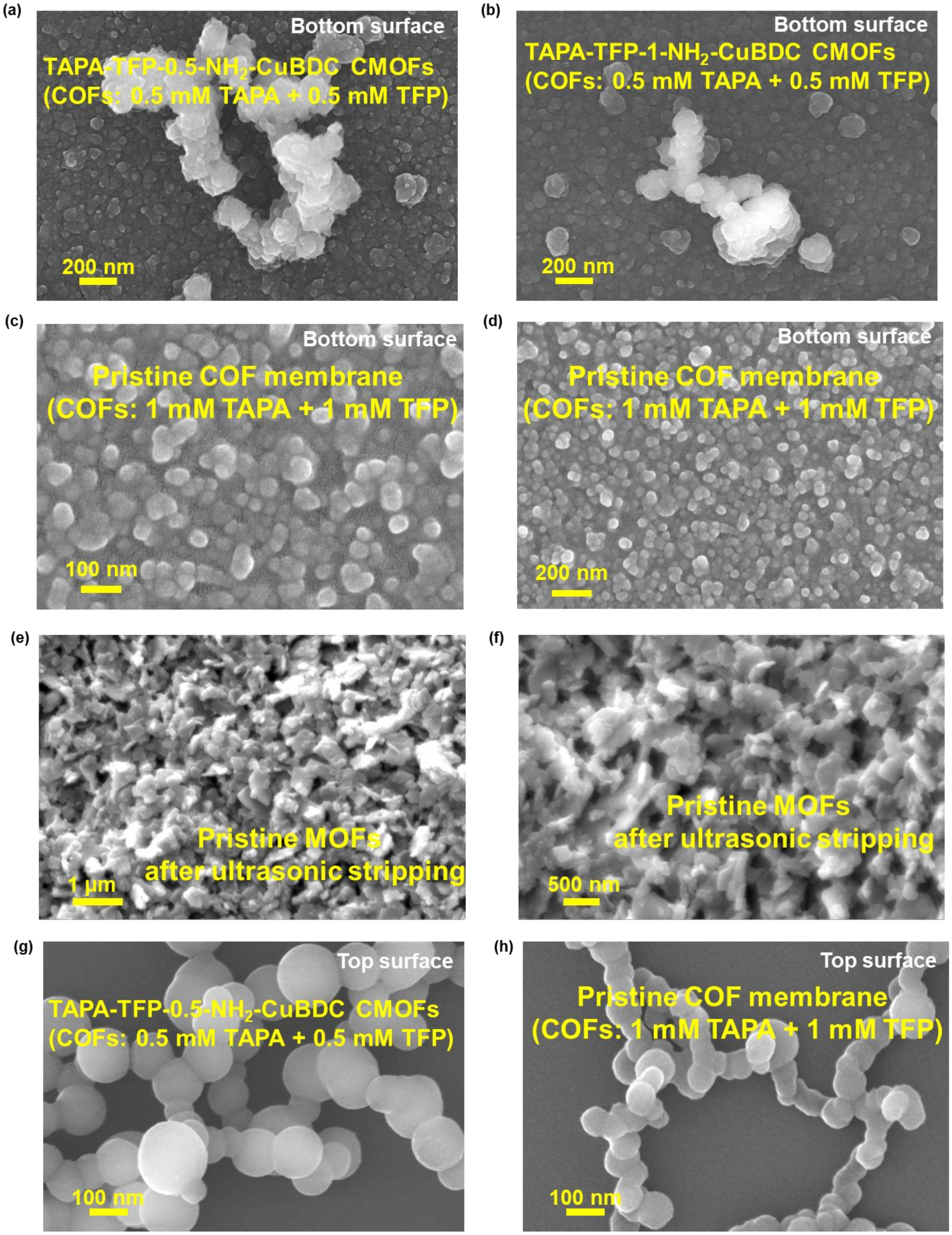

Supplementary Figure 3. SEM images of the nanoparticles on the surfaces of TAPA-TFP-*x*-NH_2-CuBDC CMOF composite membranes (COFs: 0.5 mM TAPA + 0.5 mM TFP) and pristine COF membrane (COFs: 1 mM TAPA + 1 mM TFP).

Supplementary Notes: The nanoparticles on the surfaces of the pristine COF membrane and CMOF membranes are almost identical and we could not find definite MOFs on the top and bottom surfaces of CMOF membranes. Furthermore, the surfaces of pristine COF membrane (COFs: 0.5 mM TAPA + 0.5 mM TFP) are very smooth without any particles present, and another pristine COF membrane (COFs: 1 mM TAPA + 1 mM TFP) was employed to compare with the CMOF membranes.

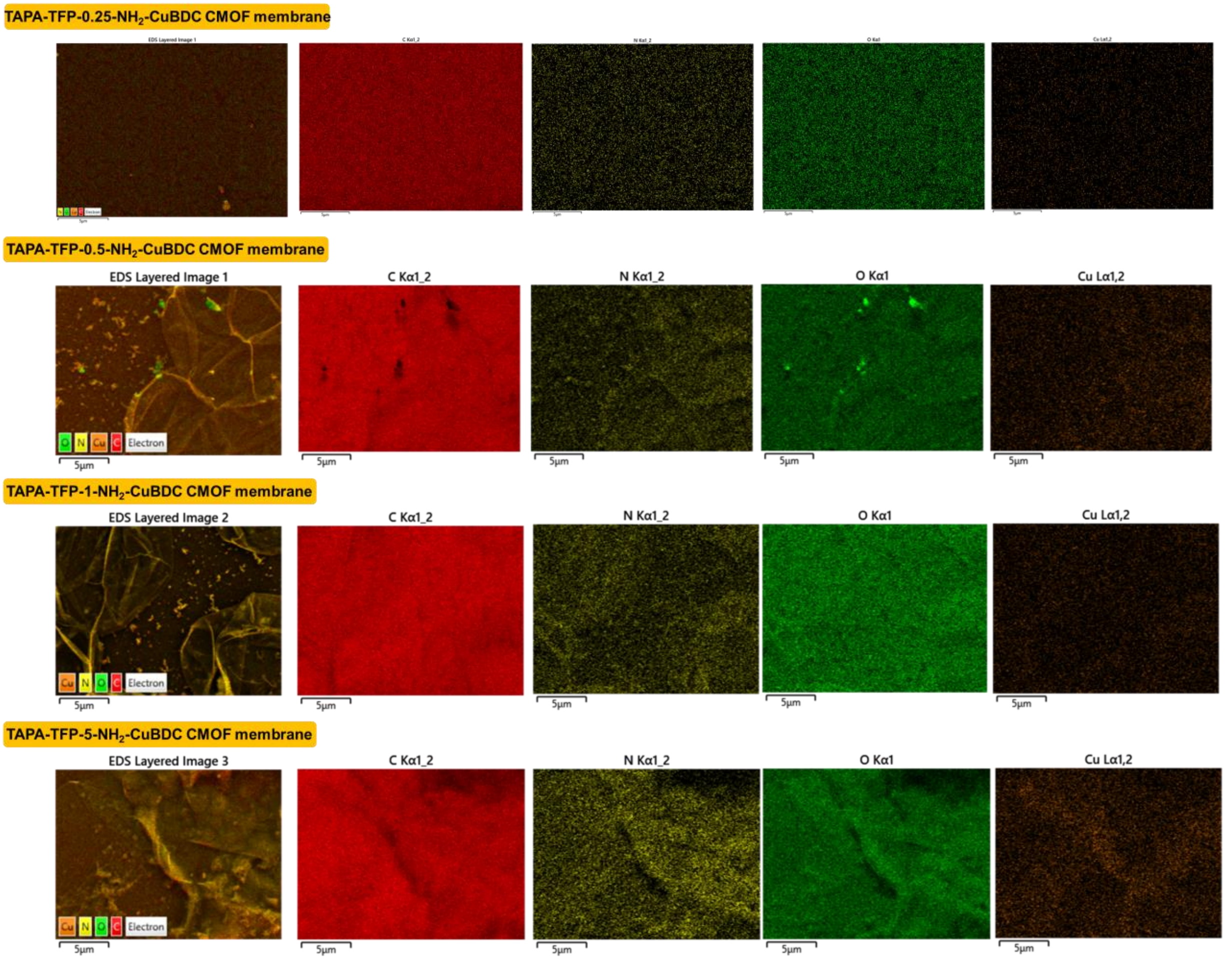

Supplementary Figure 4. EDXS mapping and elemental distributions of prepared TAPA-TFP-*x*-NH_2-CuBDC CMOF composite membranes (COFs: 0.5 mM TAPA + 0.5 mM TFP).

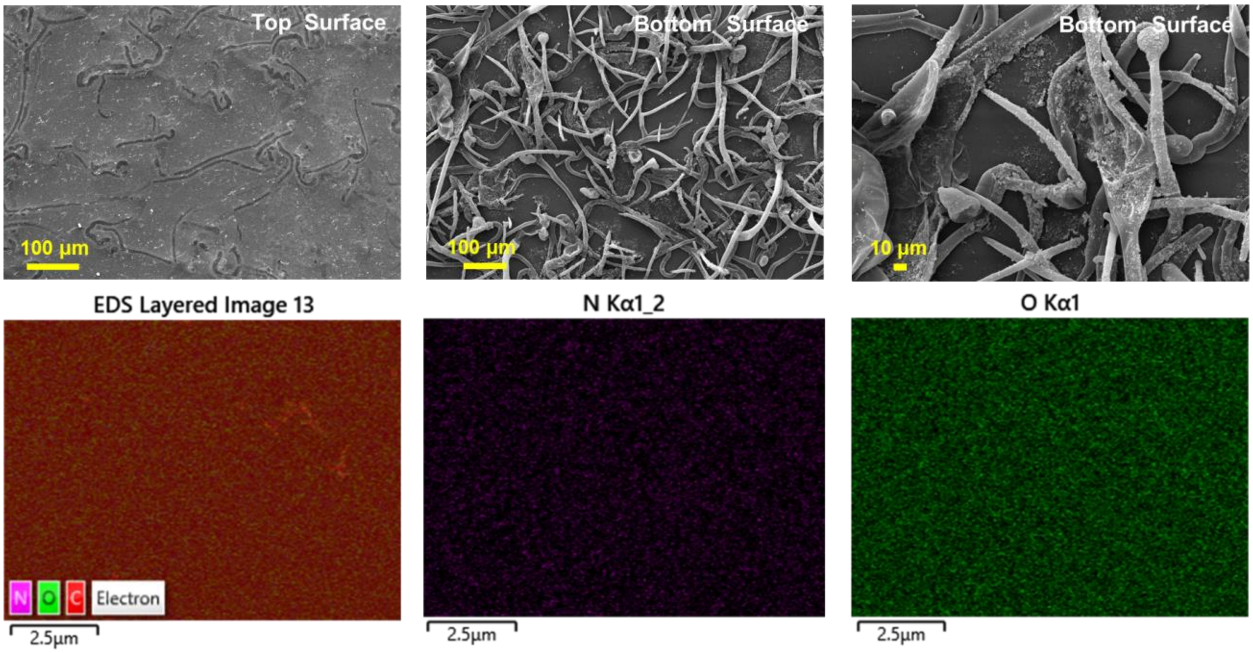

Supplementary Figure 5. SEM images and EDXS mapping of the prepared membrane referring to the synthesis method of CMOF membranes, except that NH_2-BDC is not added to the underlying oil phase (COFs: 2 mM TAPA + 2 mM TFP).

Supplementary Notes: Based on the SEM images and EDXS mapping, it is concluded that isolated copper ions from $Cu(NO_3)_2$ cannot coordinate with COFs. The reasons are as follows in two aspects: (1) No copper signal was detected on the membrane (COF+$Cu(NO_3)_2$). If isolated copper ions could coordinate with COFs, their signal should theoretically have been detectable by EDXS mapping (Note that EDXS mapping is sensitive to the concentration of the element being detected, and herein the thicker pristine membrane that may trap more copper ions was chosen in order to maximize the detection of the copper ion signal.). (2) The long vermicular nanotubes distributed on the bottom surface of the prepared membrane remained in a plump state. If isolated copper ions could coordinate with COFs, the generated heat would have caused the plump COFs to become shrunken or even broken (like the nanotubes distributed on CMOF or MCOF membranes in **Supplementary Figure 2** or **Supplementary Figure 17**).

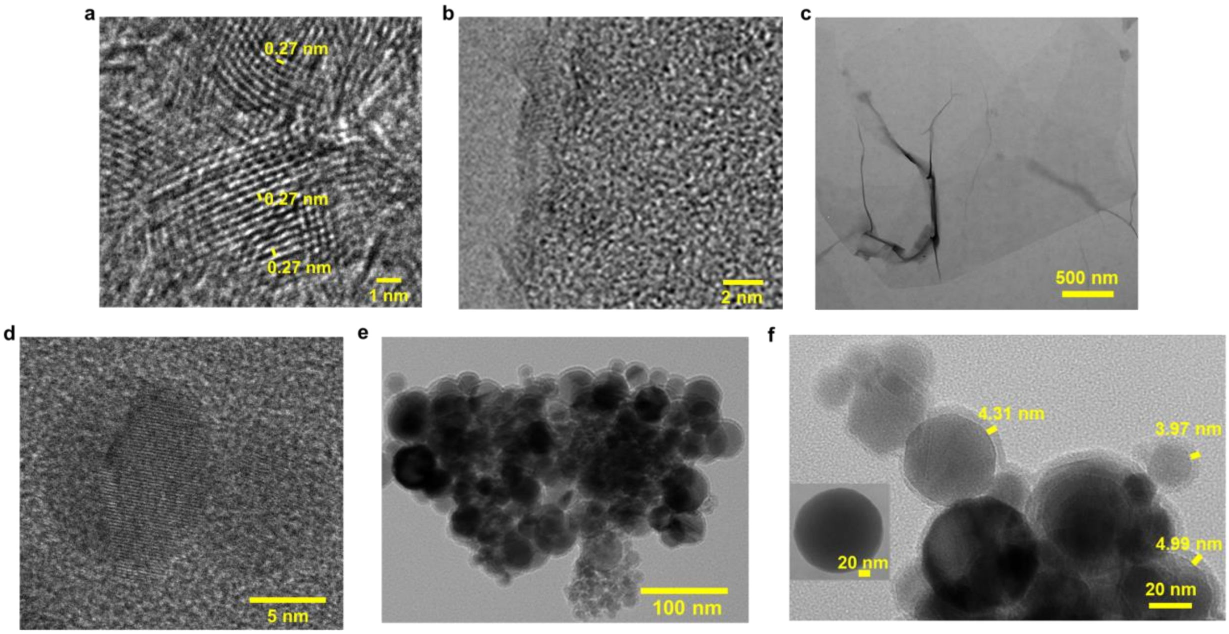

Supplementary Figure 6. TEM images of pristine MOFs (0.25-NH_2-CuBDC MOFs) (a), the pristine COF membrane (0.5 mM TAPA + 0.5 mM TFP) (b) and the TAPA-TFP-0.25-NH_2-CuBDC CMOF composite membrane (COFs: 0.5 mM TAPA + 0.5 mM TFP) (c and d show different magnification levels.). TEM images of the powders collected from the petri dish for preparing TAPA-TFP-0.25-NH_2-CuBDC CMOF composite membrane (e and f show different magnification levels.).

Supplementary Notes: For **Supplementary Figure 6f**, inset is the TEM image of pristine COF powders. The obtained CMOF powders exhibit an exquisite core-shell heterostructure, and the MOF shell thickness is approximately 4 nm.

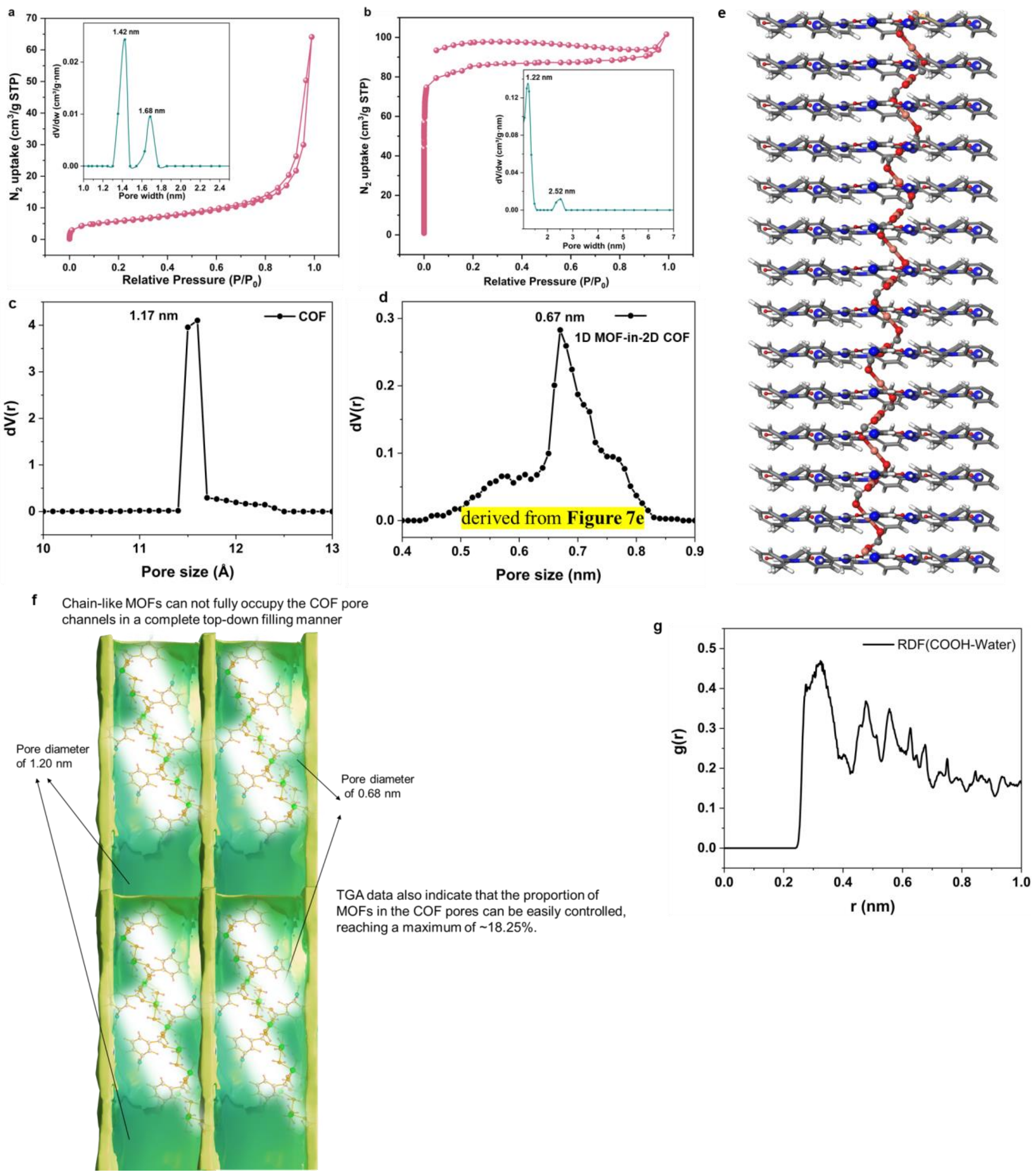

Supplementary Figure 7. Pore size distributions (insets) and Brunauer-Emmett-Teller (BET) surface areas of pristine MOFs (a) and COF membranes (b). Simulated pore size distributions of pristine COFs (c) and 1D MOF-in-2D COF hetero-structure (derived from **Supplementary Figure 7e**) (d). The simulated 1D MOF-in-2D COF hetero-structure (Some atoms are omitted for clarity.)

(e). Schematic diagram of CMOF pore channels (f). Radial distribution function (RDF) of oxygen in water molecules around -COOH groups in CMOF channels (The RDF peak value is relatively low, indicating that the probability of water molecules appearing around -COOH groups in CMOF channels is low. Therefore, a stable hydration layer cannot be formed.) (g).

Supplementary Notes: The pristine MOFs and COF membrane respectively exhibit sharp peaks centered at 1.42 and 1.22 nm. The Brunauer-Emmett-Teller surface areas of the pristine COF membrane and MOFs are calculated to be 307.41 and 19.71 m^2 g^{-1}, respectively, based on the 77 K N_2 sorption results.

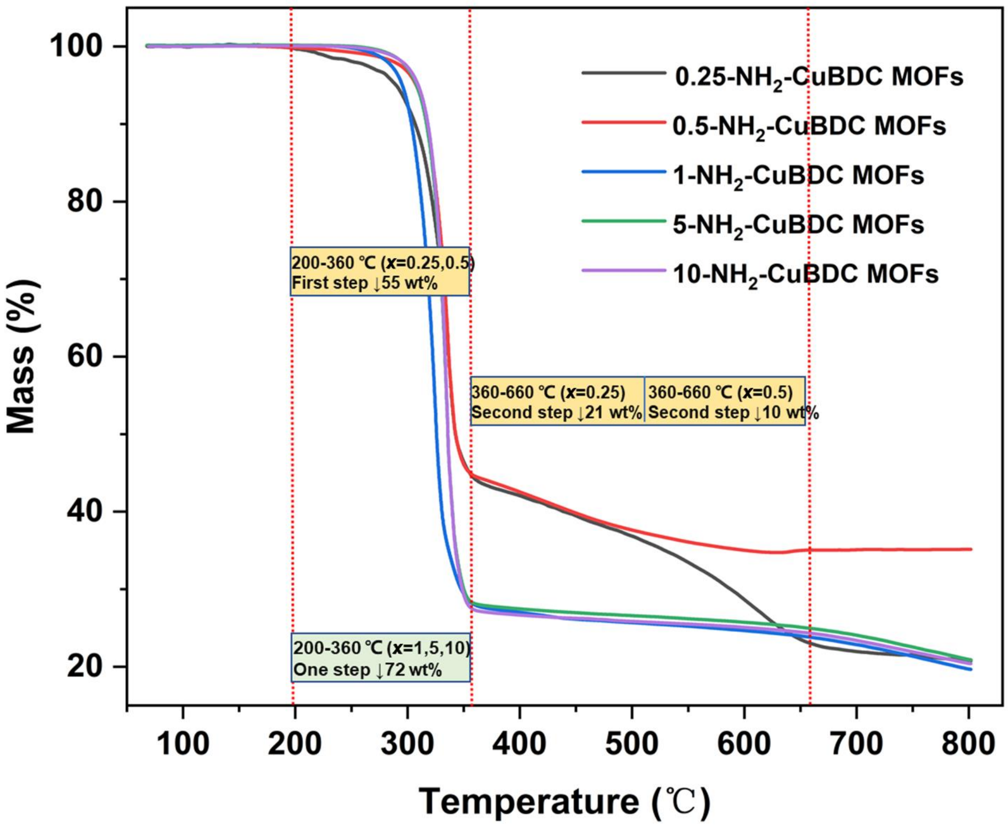

42

Supplementary Figure 8. TGA data of synthesized pristine MOFs.

Supplementary Notes: For the 1-NH_2-CuBDC, 5-NH_2-CuBDC and 10-NH_2-CuBDC MOFs, a 72% weight loss occurs at 200-360 °C. However, the weight loss in 0.25-NH_2-CuBDC and 0.5-

NH_2-CuBDC MOFs occurs in two steps: a 55% weight loss in the first stage (200-360 °C) and a second weight loss of 21% or 10% (360-660 °C).

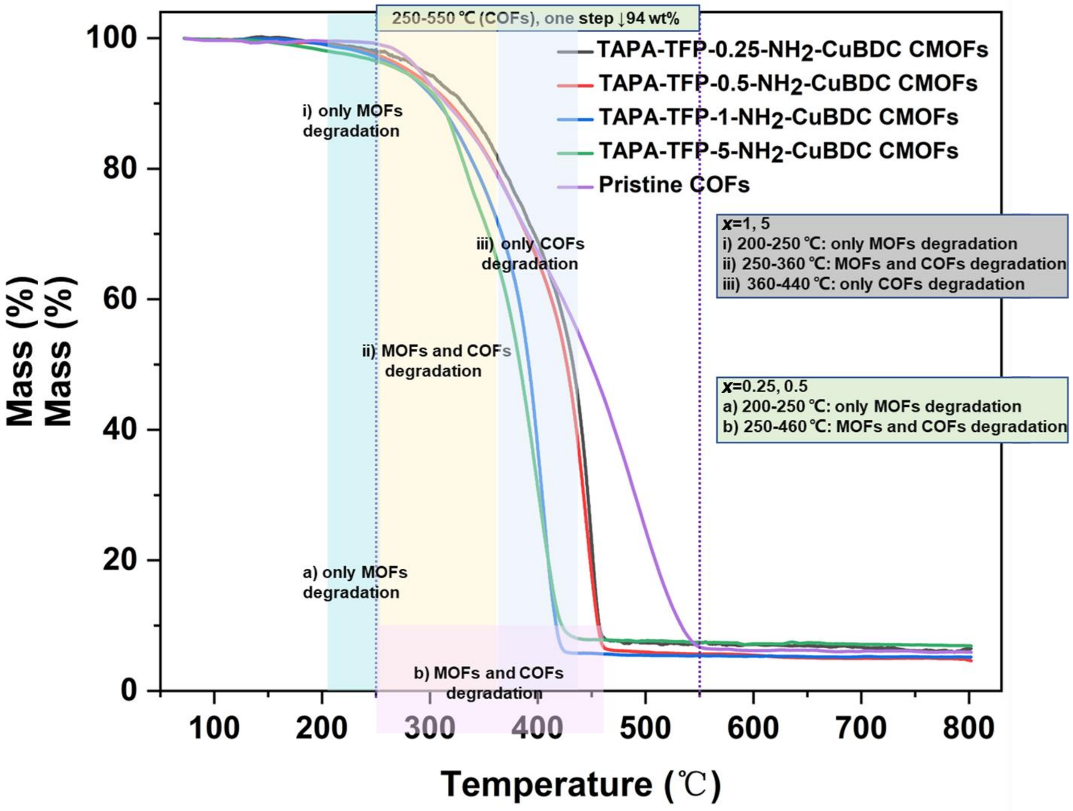

Supplementary Figure 9. TGA data of synthesized CMOF composite membranes (COFs: 0.5 mM TAPA + 0.5 mM TFP).

Supplementary Notes: Pristine COFs have a 94% weight loss at 250-550 °C. For the TAPA-TFP-1-NH_2-CuBDC and TAPA-TFP-5-NH_2-CuBDC CMOFs, the weight loss occurs in three steps: i) 200-250 °C, only MOFs degradation, ii) 250-360 °C: MOFs and COFs degradation, iii) 360-440 °C: only COFs degradation. According to the weight loss in the third stage, the weight loadings of MOFs in TAPA-TFP-1-NH_2-CuBDC and TAPA-TFP-5-NH_2-CuBDC CMOFs are respectively ~7.44% and ~18.25%. For the TAPA-TFP-0.25-NH_2-CuBDC and TAPA-TFP-0.5-NH_2-CuBDC

CMOFs, the weight loss occurs in two steps: i) 200-250 °C, only MOFs degradation, ii) 250-460 °C: MOFs and COFs degradation. It is really difficult to determine the MOFs loadings with the available results, but the loadings should be below 7.44%.

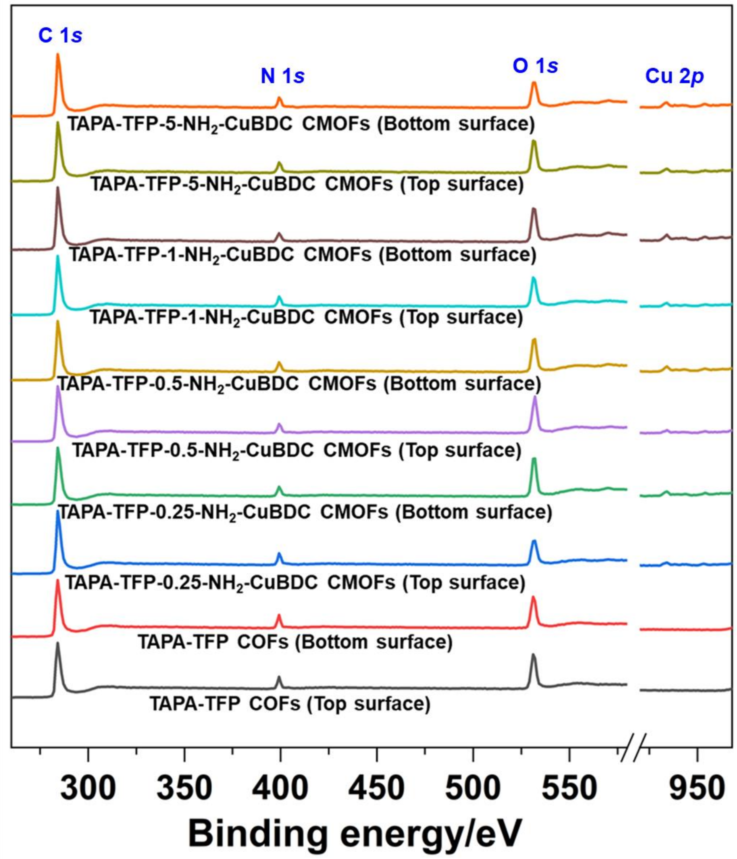

Supplementary Figure 10. XPS spectra of synthesized CMOF composite membranes (COFs: 0.5 mM TAPA + 0.5 mM TFP). The Cu signals confirm the successful introduce of MOFs in CMOFs.

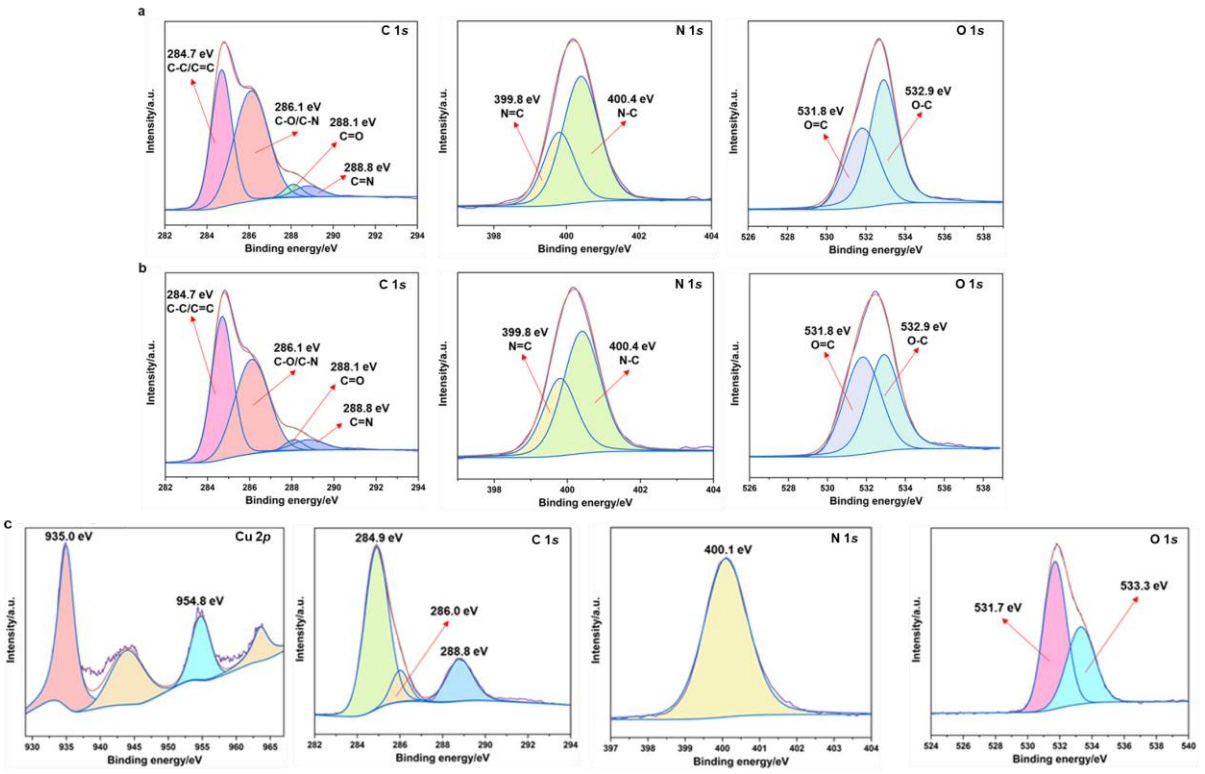

Supplementary Figure 11. XPS spectra of the top (a) and bottom surfaces (b) of TAPA-TFP COF membranes (COFs: 0.5 mM TAPA + 0.5 mM TFP). (c) XPS spectra of pristine MOFs.

Supplementary Notes: There is no shift in the binding energy between the top and bottom surfaces of the pristine COF membrane. The C 1*s* spectrum at 288.8, 288.1, 286.1 and 284.7 eV can be assigned to the C in C=N, C=O, C-O/C-N, and C=C/C–C, separately. The N 1*s* spectrum at 400.4 eV and 399.8 eV can be ascribed to the N in N-C and N=C, respectively. The O 1*s* spectrum at 532.9 eV and 531.8 eV can be ascribed to the O in O-C and O=C, respectively. For the XPS spectra of pristine MOFs. The binding energy of Cu 2*p* of Cu is 935.0 eV and 954.8 eV. The C 1*s* spectrum at 284.9 eV, 286.0 eV and 288.8 eV can be assigned to the C in C-C, C-N/C-O and C=O. The N 1*s* spectrum at 400.1 eV is ascribed to the N in N-C. The O 1*s* spectrum at 533.3 eV and 531.7 eV can be ascribed to the O in O-C and O=C, respectively.

TAPA-TFP-0.25-NH_2-CuBDC CMOFs (Top surface)

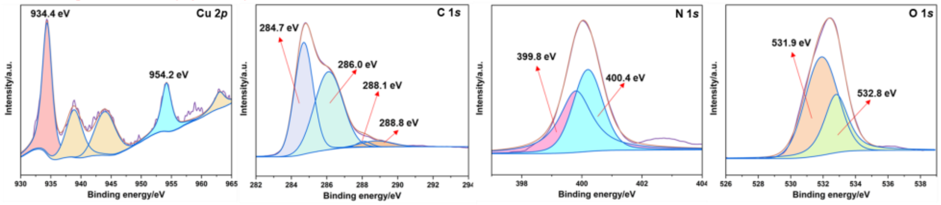

TAPA-TFP-0.25-NH_2-CuBDC CMOFs (Bottom surface)

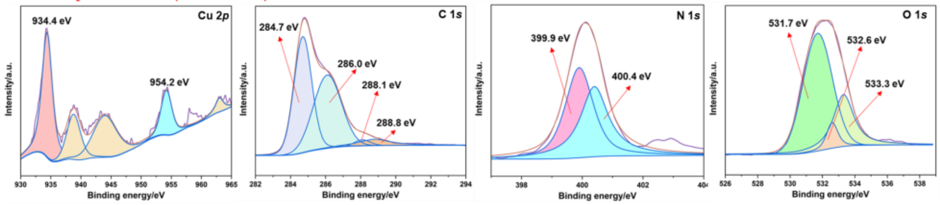

TAPA-TFP-0.5-NH_2-CuBDC CMOFs (Top surface)

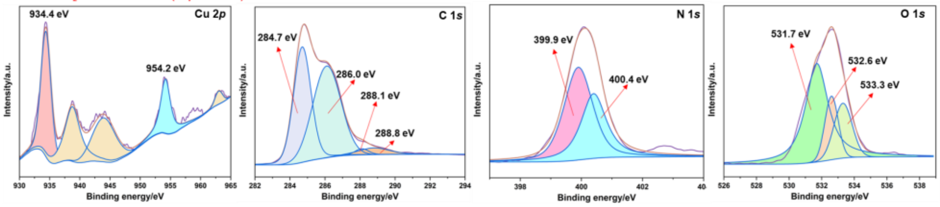

TAPA-TFP-0.5-NH_2-CuBDC CMOFs (Bottom surface)

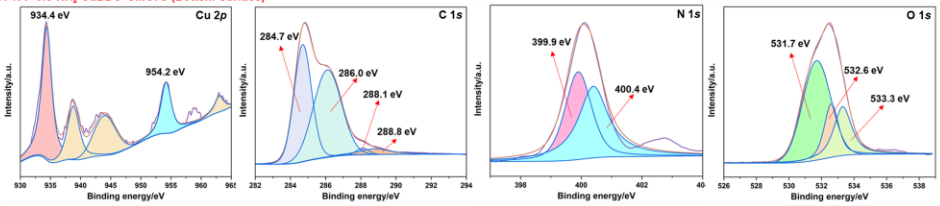

TAPA-TFP-1-NH_2-CuBDC CMOFs (Top surface)

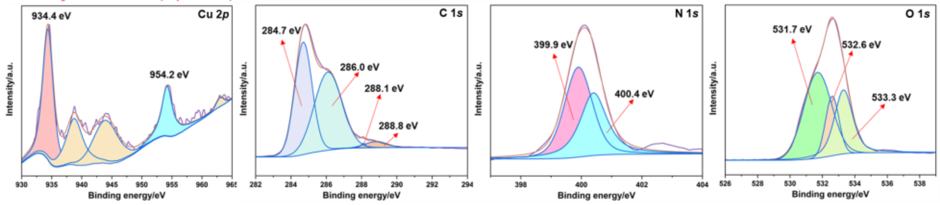

TAPA-TFP-1-NH_2-CuBDC CMOFs (Bottom surface)

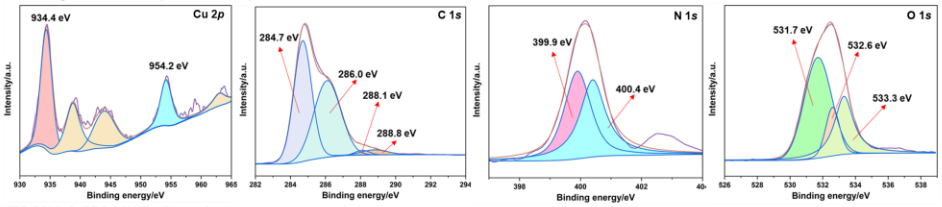

TAPA-TFP-5-NH_2-CuBDC CMOFs (Top surface)

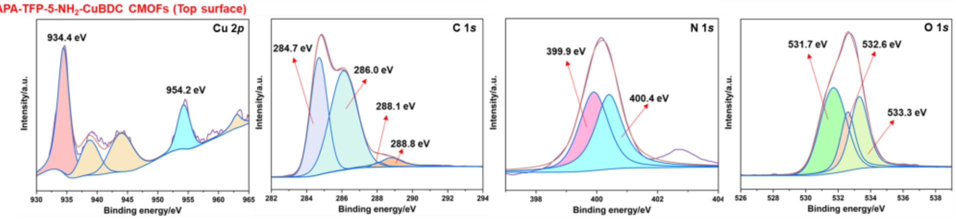

TAPA-TFP-5-NH_2-CuBDC CMOFs (Bottom surface)

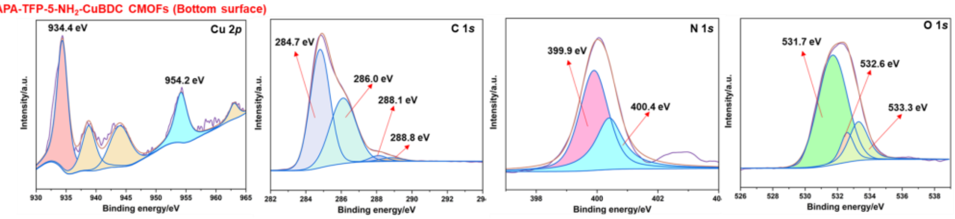

Supplementary Figure 12. XPS spectra of synthesized CMOF composite membranes (COFs: 0.5 mM TAPA + 0.5 mM TFP).

Supplementary Notes: There is little difference in the XPS spectra between the top surface and bottom surface of CMOF composite membranes. Furthermore, with the increase of MOF ligand concentration, the XPS spectra hardly changed. Taking the TAPA-TFP-5-NH_2-CuBDC CMOF composite membrane as a case, the C 1*s* spectrum at 288.8, 288.1 and 284.7 eV can be separately assigned to the C in C=N, C=O and C=C/C–C in COFs and the C 1*s* spectrum at 286.0 eV can be assigned to the C in C-N/C-O in MOFs. The N 1*s* spectrum at 400.4 eV can be ascribed to the N in N-C in COFs. Another peak is centred at 399.9 eV, which is greater than that of the pristine COFs (399.8 eV) and smaller than that of pristine MOFs (400.1 eV). This may be the result of electron transfer between MOFs and the N atoms from COFs due to the interaction [1]. Most importantly, the binding energy of Cu 2*p* of Cu in MOFs is 935.0 eV and 954.8 eV. It shifts to the lower binding energy of 934.4 eV and 954.2 eV in the CMOF membranes, implying the coordination interactions between Cu(II) in MOFs and the N in COFs [2-4].

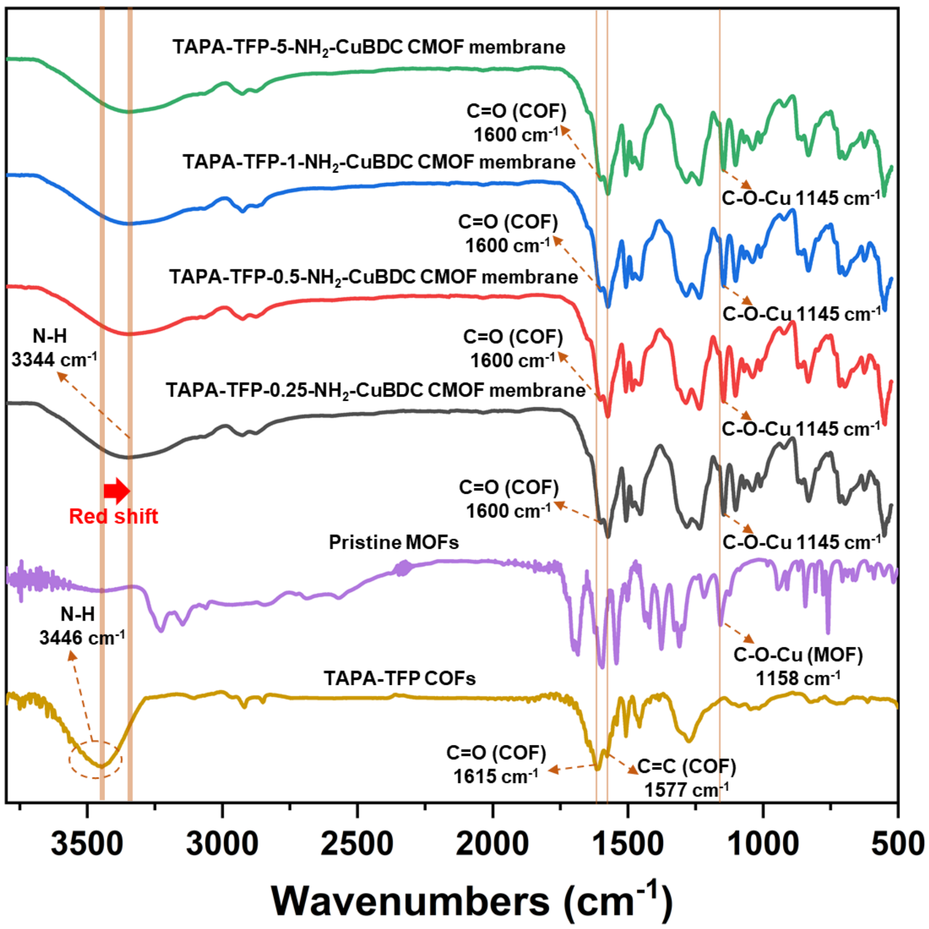

Supplementary Figure 13. ATR-FTIR spectra of synthesized CMOF composite membranes (COFs: 0.5 mM TAPA + 0.5 mM TFP).

Supplementary Notes: The TAPA-TFP-*x*-NH$_2$-CuBDC CMOFs exhibit nearly consistent spectra. Compared with pristine MOFs, the characteristic peaks from C-O-Cu stretching vibrations of TAPA-TFP-*x*-NH$_2$-CuBDC CMOFs shift from 1158 cm^{-1} to 1145 cm^{-1}, demonstrating the change of the MOF coordination environment [4]. Compared with pristine COFs, the peak from N-H stretching vibrations shifts from 3446 cm^{-1} to 3344 cm^{-1} due to the coordination between the -NH groups in COFs and the Cu centers from MOFs.

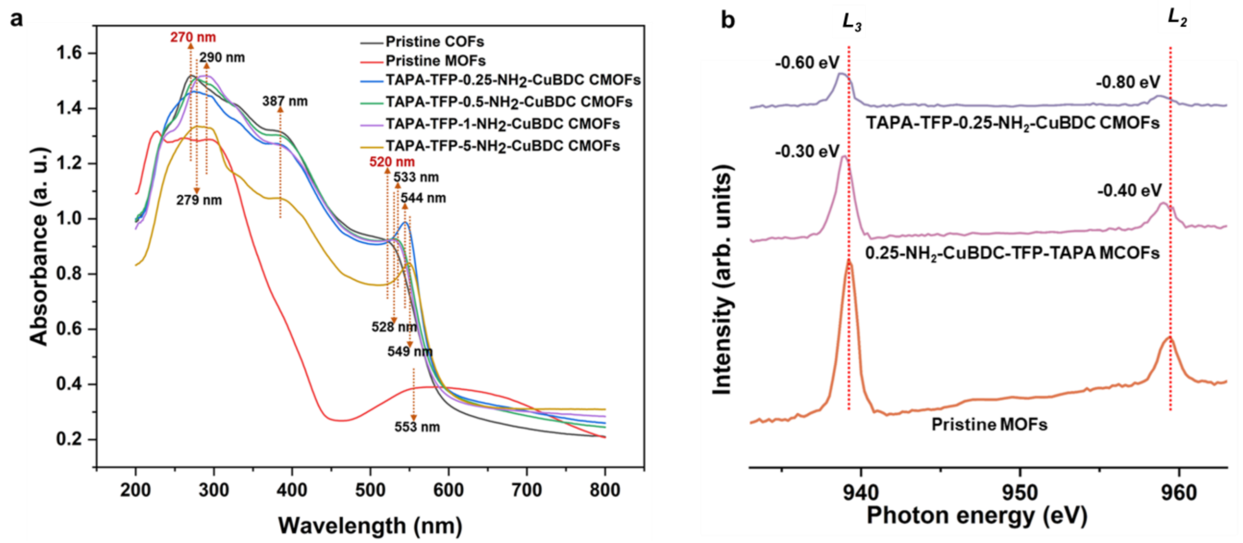

Supplementary Figure 14. (a) UV-Vis-NIR spectra of synthesized CMOF composite membranes (COFs: 0.5 mM TAPA + 0.5 mM TFP). (b) Cu $L_{3,2}$-edge XANES spectra of pristine MOFs, TAPA-TFP-0.25-NH_2-CuBDC CMOFs, and 0.25-NH_2-CuBDC-TAPA-TFP MCOFs (COFs: 0.5 mM TAPA + 0.5 mM TFP).

Supplementary Notes: The pristine COF membrane mainly shows three absorption bands centered at 270 nm, 387 nm and 520 nm. After the introduce of MOFs, different degrees of red-shift are observed in the CMOF composite membranes, which is attributed to the coordination interaction between MOFs and COFs, resulting in a smaller vibration frequency of the chemical bonds. Especially, the TAPA-TFP-0.25-CuBDC CMOF and TAPA-TFP-5-NH_2-CuBDC CMOF composite membranes show stronger light absorption at approximately 546 nm, suggesting that there are stronger coordination interactions and the membranes may exhibit outstanding performances. Furthermore, XANES spectra of Cu *L* edge is a transition from 2*p* to 3*d* state. The peak shifts of Cu L_3 ($2p_{3/2}$) and Cu L_2 ($2p_{1/2}$) edges are attributed to the changes in the MOF coordination environment. It should be noted that the coordination interaction may be very weak in the MCOFs due to the very slight negative shift and the negative shift of CMOFs is 2-fold that

of MCOFs. We have also attempted to confirm this Cu-O to Cu-N coordination transition using Cu *K*-edge synchrotron-based extended X-ray absorption fine structure (EXAFS) spectroscopy. However, due to their extremely close peaks, this coordination change cannot be accurately identified.

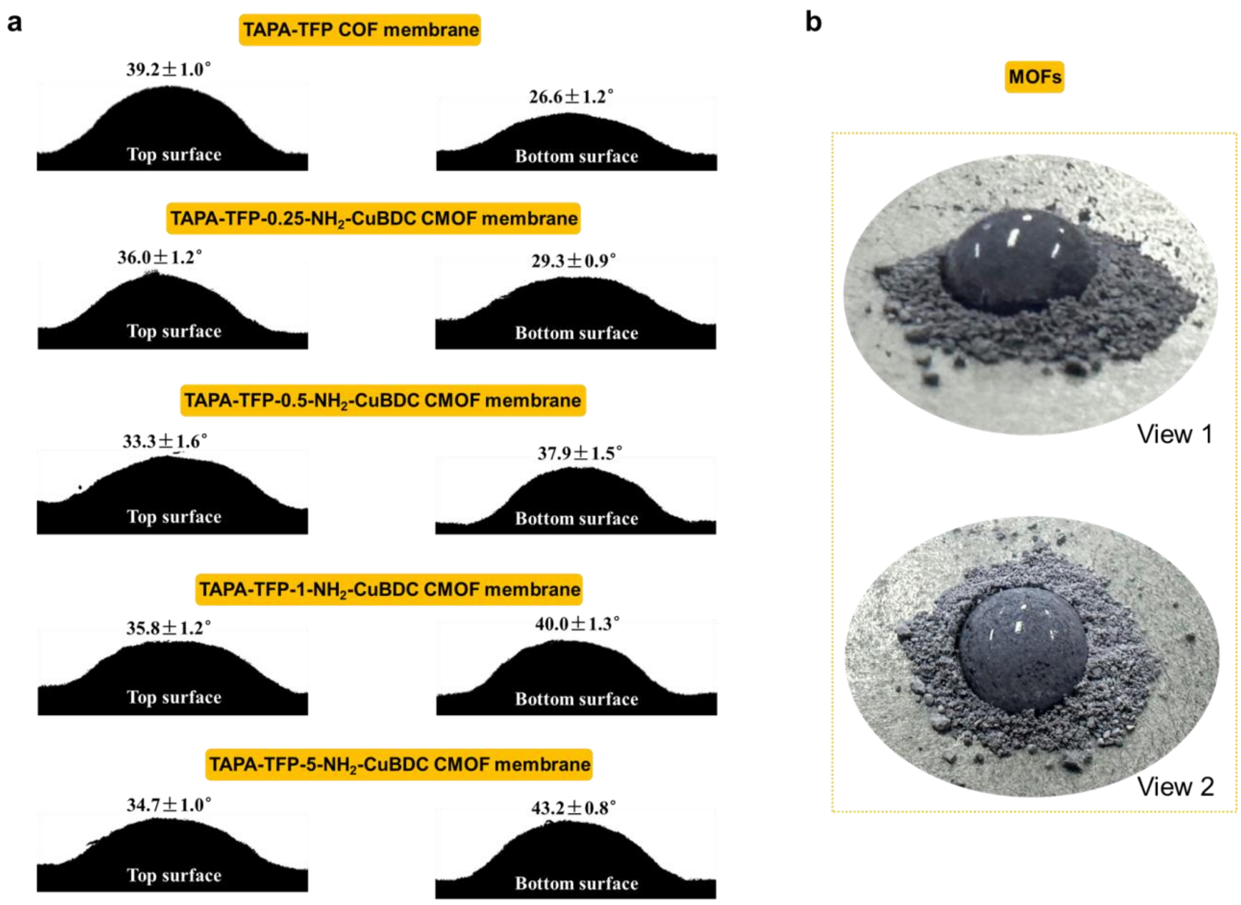

Supplementary Figure 15. Surface wettability of prepared TAPA-TFP-*x*-NH_2-CuBDC CMOF composite membranes and pristine MOFs (COFs: 0.5 mM TAPA + 0.5 mM TFP) (a). (b) Electronic photographs of the moment when a single drop of water lands on the surface of the pristine MOF powders (Two shooting perspectives result in view 1 and view 2).

Supplementary Notes: Compared with pristine COF membrane, the top surface wettability of TAPA-TFP-*x*-NH_2-CuBDC CMOF composite membrane barely changed, and its bottom surface is slightly more hydrophobic, which results from the introduce of MOFs.

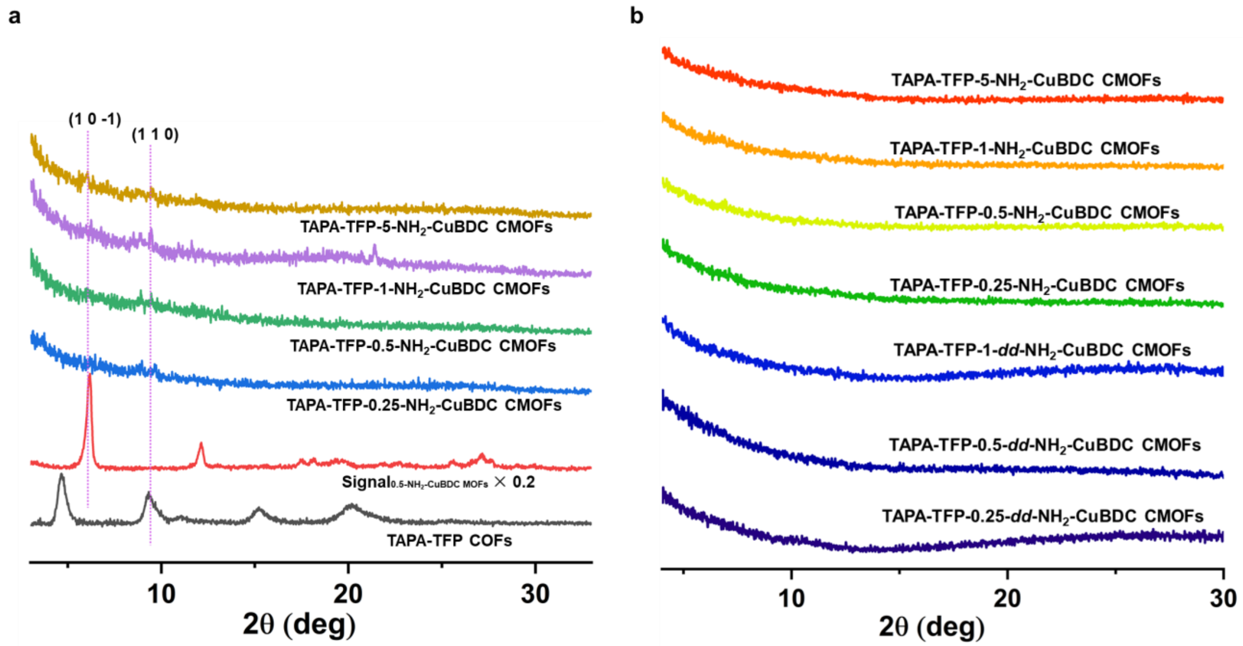

Supplementary Figure 16. XRD patterns of CMOF composite membranes. a, COFs: 1 mM TAPA + 1 mM TFP. b, COFs: 2 mM TAPA + 2 mM TFP.

Supplementary Notes: It should be noted that the thicker the pristine COF membrane is, the more easily the XRD peak of MOFs is covered.

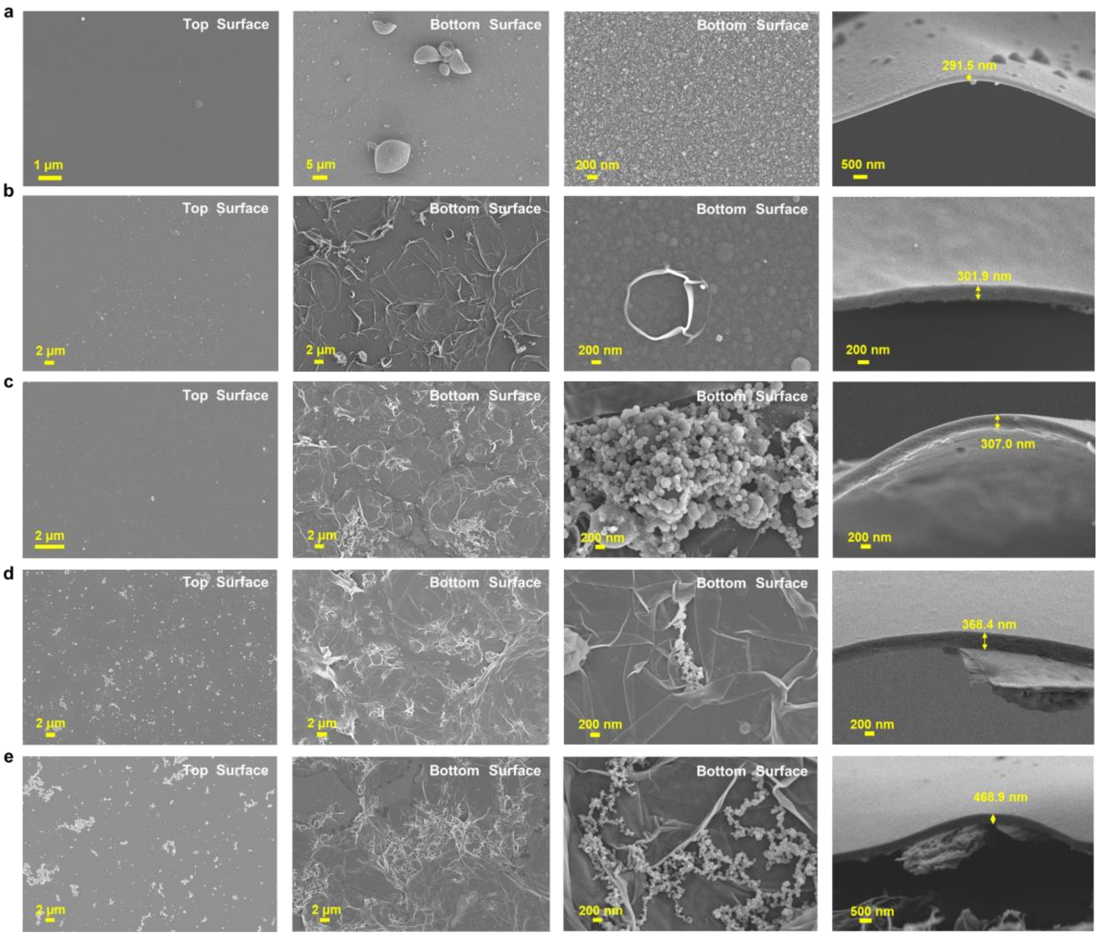

Supplementary Figure 17. SEM images of the pristine COF membrane (1 mM TAPA + 1 mM TFP) (a), TAPA-TFP-0.25-NH_2-CuBDC CMOF composite membrane (b), TAPA-TFP-0.5-NH_2-CuBDC CMOF composite membrane (c), TAPA-TFP-1-NH_2-CuBDC CMOF composite membrane (d) and TAPA-TFP-5-NH_2-CuBDC CMOF composite membranes (e).

Supplementary Notes: Similarly, we failed to find definite MOFs on the membrane surface. The TAPA-TFP-*x*-NH_2-CuBDC CMOF membrane shows a Janus morphology and its bottom surface is evenly distributed with larger broken COF vesicles. Furthermore, with the increase of MOF ligand concentration, the thickness of the CMOF membrane gradually increases. The pristine COF membrane shows a thickness of 291.5 nm, while the TAPA-TFP-0.25-NH_2-CuBDC, TAPA-TFP-0.5-NH_2-CuBDC, TAPA-TFP-1-NH_2-CuBDC and TAPA-TFP-5-NH_2-CuBDC CMOF membranes respectively exhibit the thicknesses of 301.9 nm, 307.0 nm, 368.4 nm and 468.9 nm.

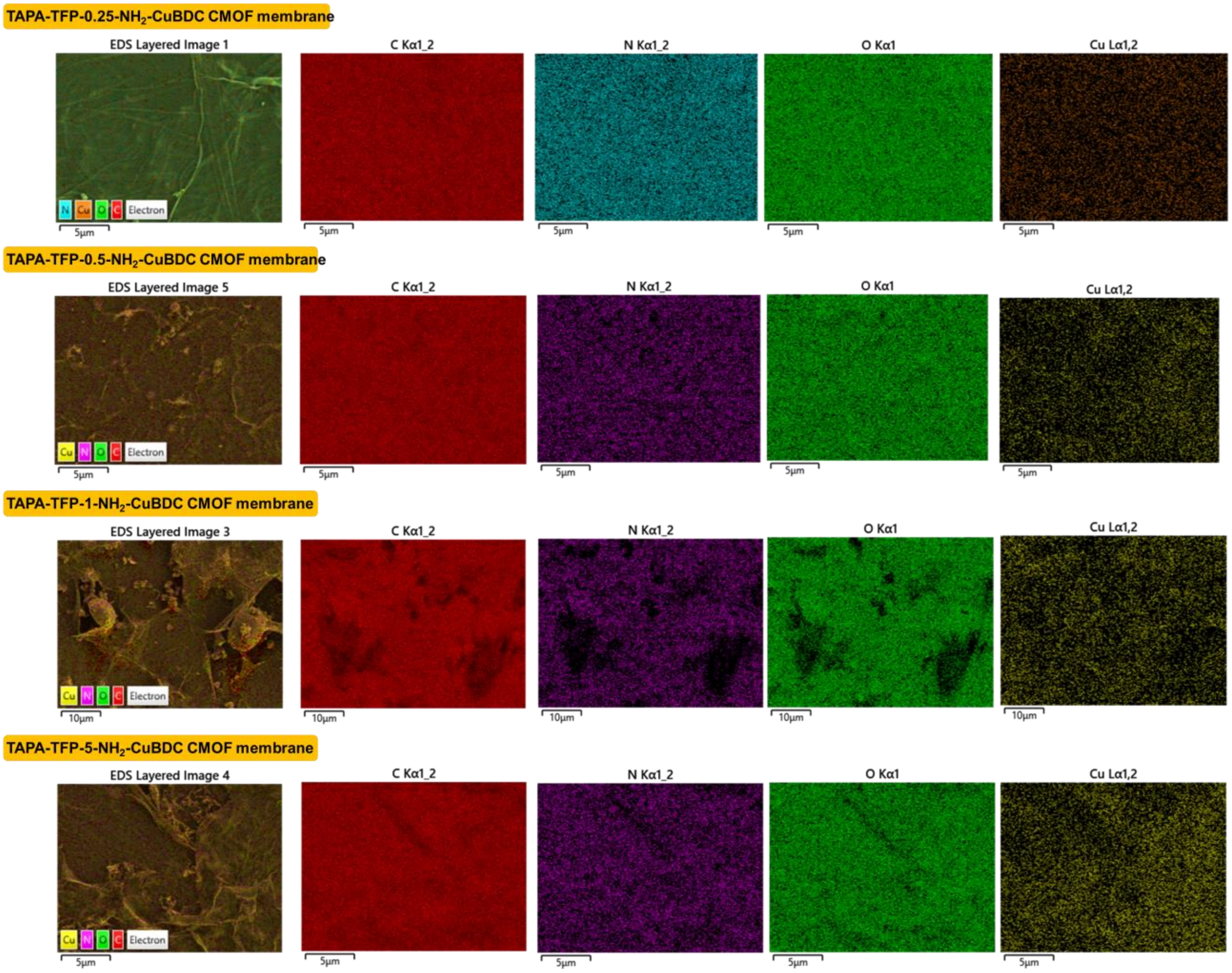

64

Supplementary Figure 18. EDXS mapping and elemental distributions of prepared TAPA-TFP-*x*-NH_2-CuBDC CMOF composite membranes (COFs: 1 mM TAPA + 1 mM TFP).

Supplementary Notes: The Cu signals from EDXS confirm a good distribution of MOFs in the CMOF composite membranes.

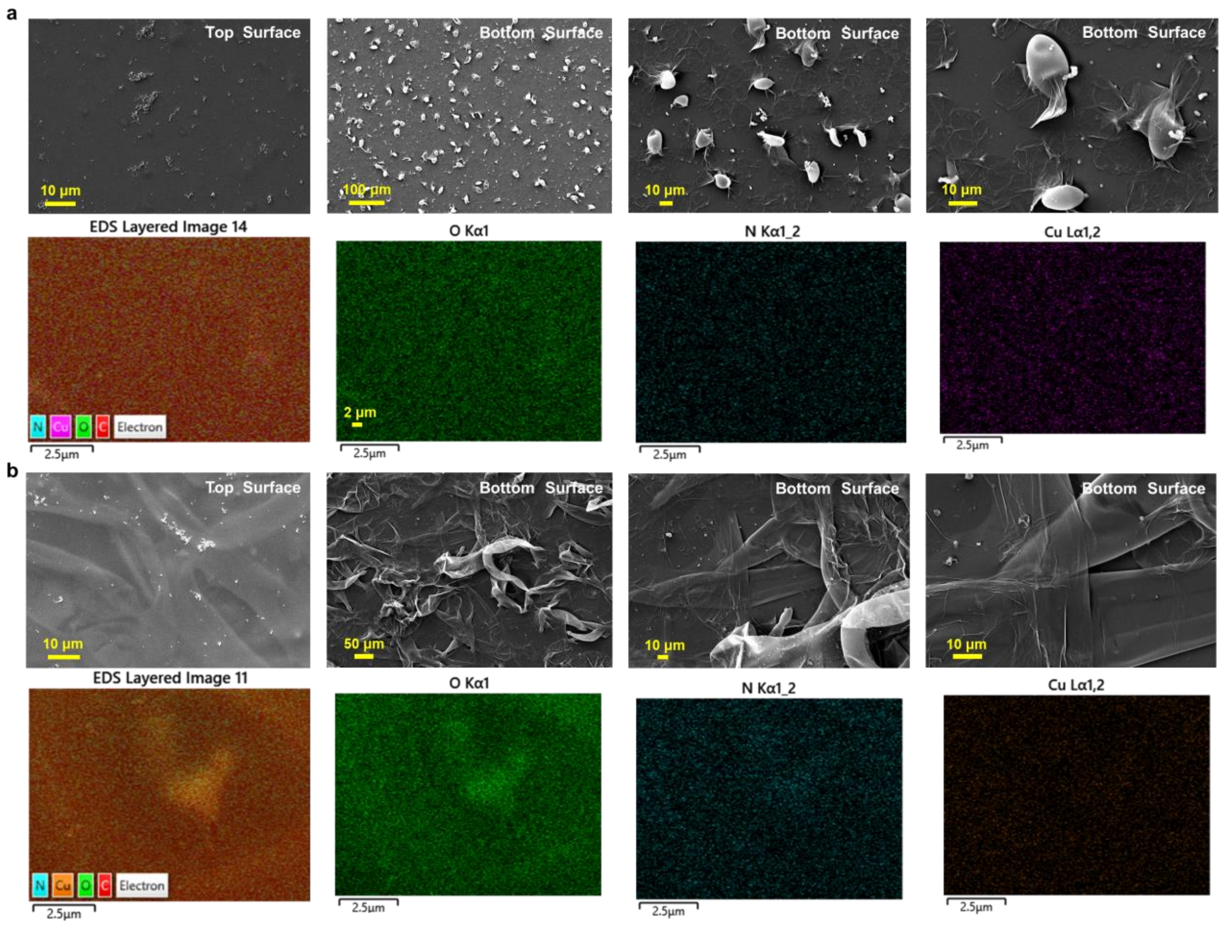

Supplementary Figure 19. SEM images and EDXS mapping of prepared TAPA-TFP-0.25-NH_2-CuBDC CMOF composite membranes (a) and TAPA-TFP-1-NH_2-CuBDC CMOF composite membranes (b) (COFs: 2 mM TAPA + 2 mM TFP).

Supplementary Notes: Similarly, we failed to find definite MOFs on the membrane surface. The TAPA-TFP-0.25-NH_2-CuBDC CMOF membrane shows a Janus morphology and its bottom surface is evenly distributed with semi-deflated COF vesicles. The bottom surface of TAPA-TFP-1-NH_2-CuBDC CMOF membrane is evenly distributed with long vermicular COF nanotubes that look squashed. The Cu signals from EDXS confirm a good distribution of MOFs in the CMOF composite membranes.

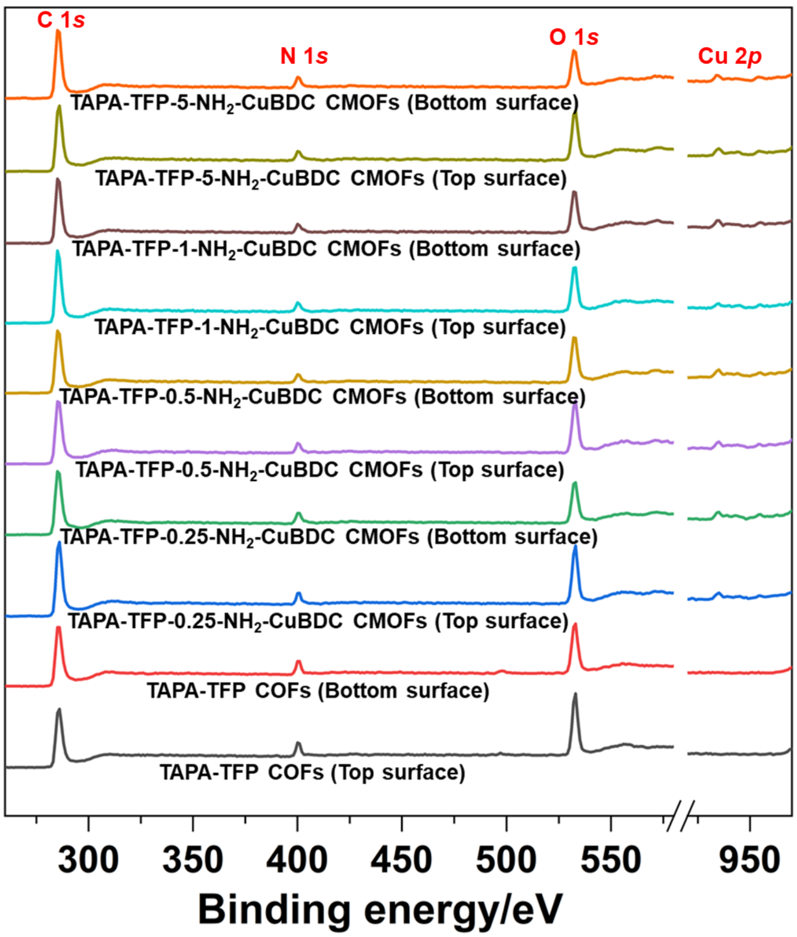

Supplementary Figure 20. XPS spectra of synthesized CMOF composite membranes (COFs: 1 mM TAPA + 1 mM TFP).

Supplementary Notes: The Cu signals confirm the successful introduce of MOFs in CMOFs.

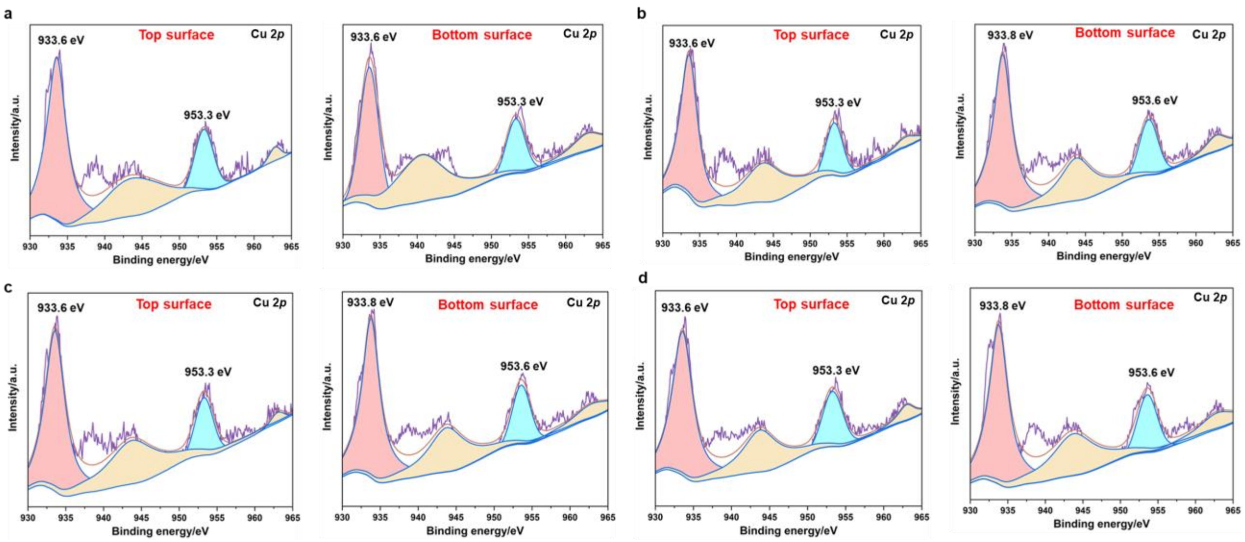

Supplementary Figure 21. Cu 2*p* spectra of TAPA-TFP-0.25-NH_2-CuBDC (a), TAPA-TFP-0.5-NH_2-CuBDC (b), TAPA-TFP-1-NH_2-CuBDC (c) and TAPA-TFP-5-NH_2-CuBDC (d) CMOF composite membranes (COFs: 1 mM TAPA + 1 mM TFP).

Supplementary Notes: The binding energy of Cu 2*p* of Cu in MOFs is 935.0 eV and 954.8 eV. It shifts to the lower binding energy of 933.6 eV and 953.3 eV in the CMOF membranes, suggesting the coordination interaction between the -NH groups in COFs and the Cu centers from MOFs.

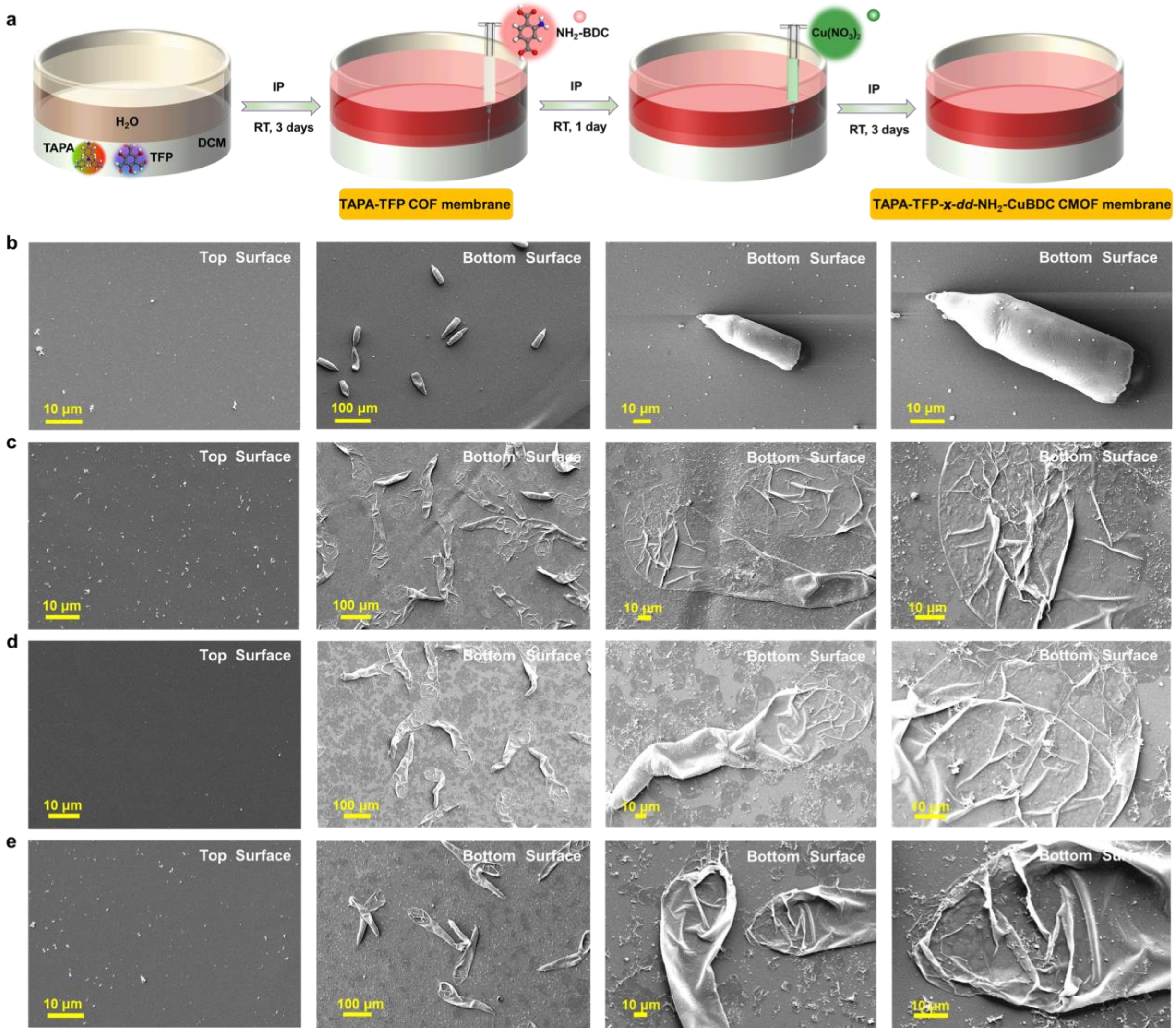

Supplementary Figure 22. Schematic synthesis illustration of TAPA-TFP-*x*-*dd*-NH_2-CuBDC CMOF composite membranes (COFs: 2 mM TAPA + 2 mM TFP) (a). SEM images of the prepared TAPA-TFP COF membrane (b), TAPA-TFP-0.25-*dd*-NH_2-CuBDC CMOF composite membrane (c), TAPA-TFP-0.5-*dd*-NH_2-CuBDC CMOF composite membrane (d) and TAPA-TFP-1-*dd*-NH_2-CuBDC CMOF composite membrane (e).

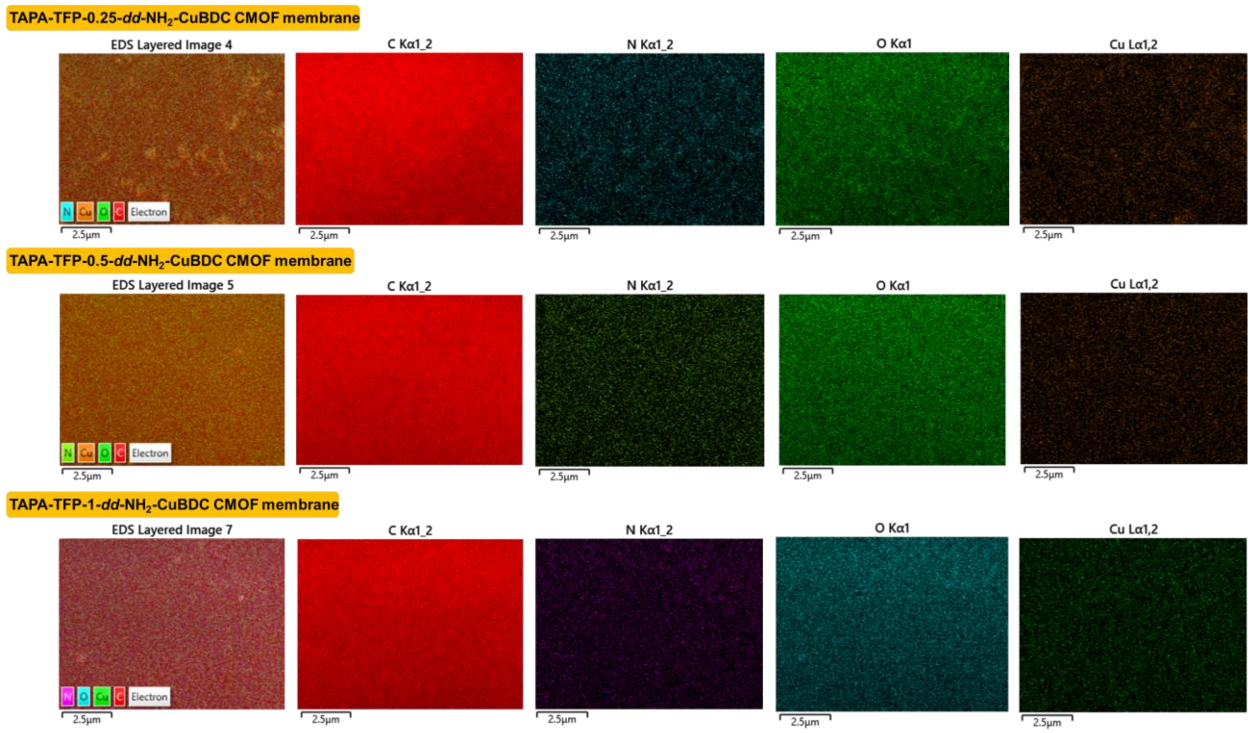

Supplementary Figure 23. EDXS mapping and elemental distributions of prepared TAPA-TFP-*x*-*dd*-NH_2-CuBDC CMOF composite membranes (COFs: 2 mM TAPA + 2 mM TFP).

Supplementary Notes: The Cu signals from EDXS confirm a good distribution of MOFs in the CMOF composite membranes.

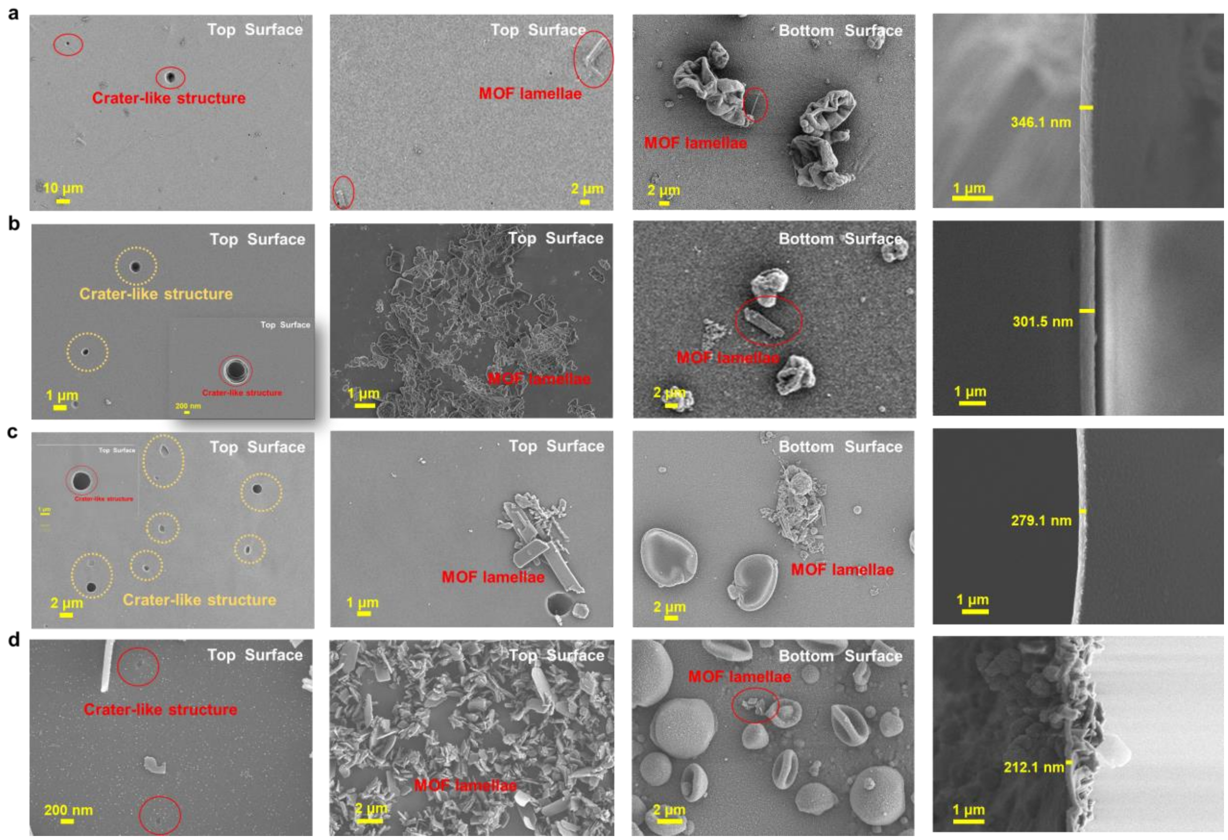

Supplementary Figure 24. SEM images of the 0.25-NH_2-CuBDC-TAPA-TFP MCOF composite membrane (a), 0.5-NH_2-CuBDC-TAPA-TFP MCOF composite membrane (b), 1-NH_2-CuBDC-TAPA-TFP MCOF composite membrane (c) and 5-NH_2-CuBDC-TAPA-TFP MCOF composite membrane (d).

Supplementary Notes: Different from the continuous thin flat COF vesicles that lie completely on the bottom surfaces of CMOF membranes, the bottom surface of MCOF membrane is sporadically distributed with thick COF hollow spheres folded inward. Interestingly, with the increase of MOF ligand concentration, the thickness of MCOF composite membrane decreases gradually. The pristine COF membrane features a thickness of 457.8 nm, while the 0.25-NH_2-CuBDC-TAPA-TFP, 0.5-NH_2-CuBDC-TAPA-TFP, 1-NH_2-CuBDC-TAPA-TFP and 5-NH_2-CuBDC-TAPA-TFP MCOF composite membranes respectively exhibit the thicknesses of 346.1 nm, 301.5 nm, 279.1 nm and 212.1 nm.

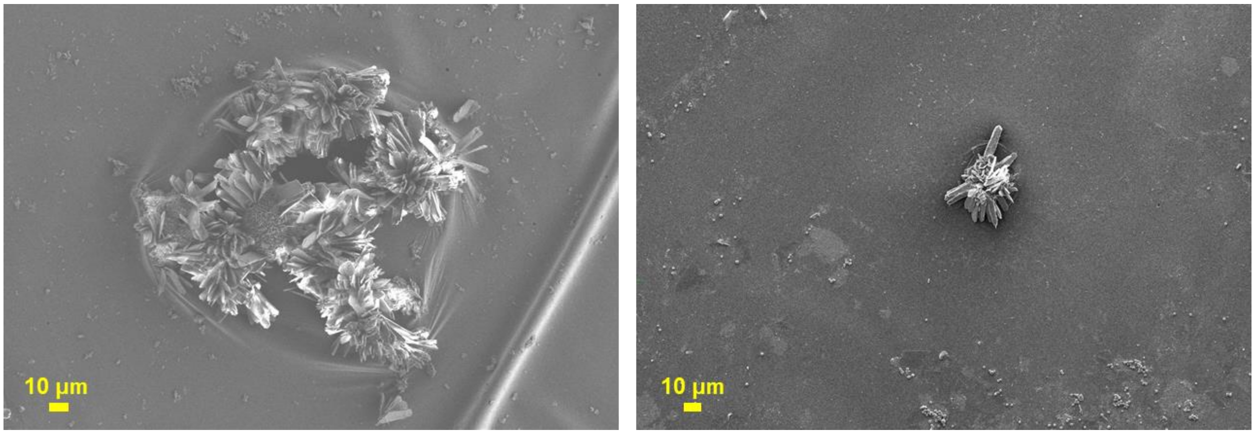

Supplementary Figure 25. SEM images of the 0.5-NH_2-CuBDC-TAPA-TFP MCOF composite membrane that has not been soaked and rinsed by methanol and water.

Supplementary Notes: It should be noted that MOF secondary particles can be observed on the surface of the composite membrane in the absence of fully rinsing and soaking.

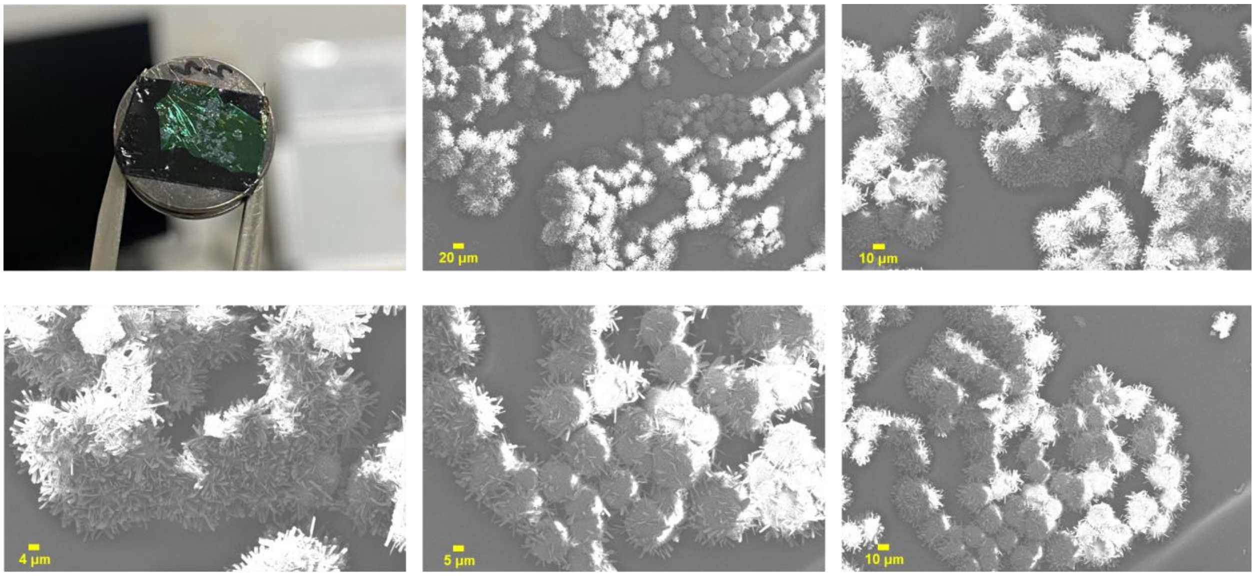

Supplementary Figure 26. Electronic and SEM images of the 5-NH_2-CuBDC-TAPA-TFP MCOF composite membrane located at the very edge of the petri dish.

Supplementary Notes: It should be noted that the membrane surface is very uniform and MOFs are visible to the naked eye only at the very edge of the petri dish. The MOFs on the membrane surface exhibit a structured hemisphere morphology, which is consistent with the pristine MOFs (5-NH_2-CuBDC MOFs).

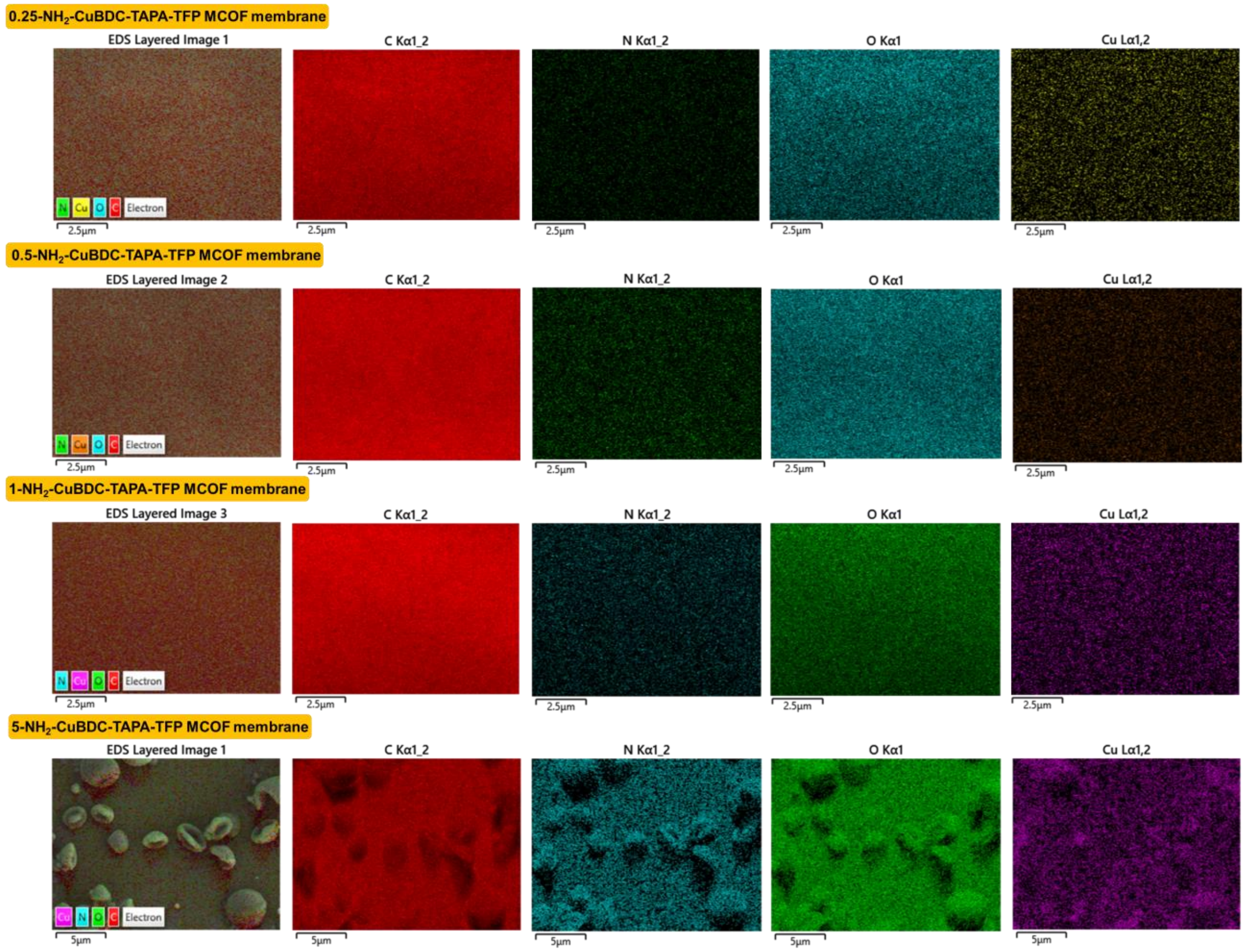

Supplementary Figure 27. EDXS mapping and elemental distributions of prepared MCOF composite membranes.

Supplementary Notes: The Cu signals from EDXS confirm a good distribution of MOFs in the MCOF composite membranes. It is worth mentioning that for the 5-NH_2-CuBDC-TAPA-TFP MCOF membrane, its top surface is almost completely covered with MOF lamellae, which may affect the mapping results, so we choose its bottom surface to conduct EDXS mapping. Note that EDXS mapping was captured where there were no visible MOFs to better observe the distribution of MOFs within the composite membrane.

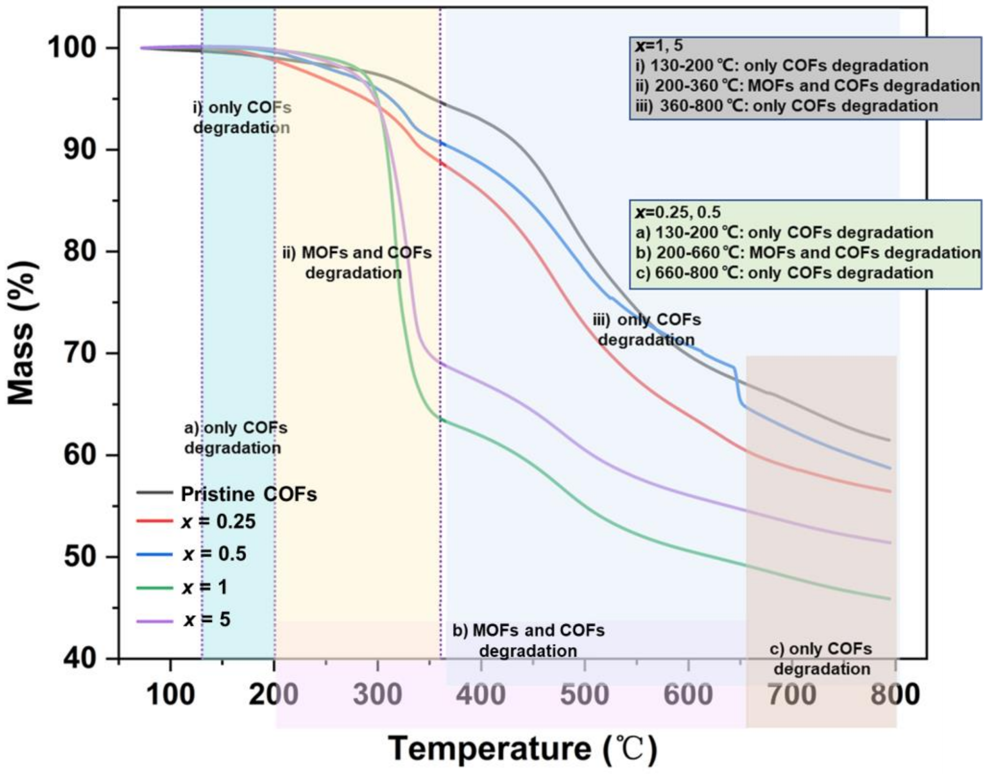

Supplementary Figure 28. TGA data of synthesized *x*-NH_2-CuBDC-TAPA-TFP MCOF composite membranes.

Supplementary Notes: For the 1-NH_2-CuBDC-TAPA-TFP and 5-NH_2-CuBDC-TAPA-TFP MCOFs, the weight loss occurs in three steps: i) 130-200 °C, only COFs degradation, ii) 200-360 °C: MOFs and COFs degradation, iii) 360-800 °C: only COFs degradation. According to the weight loss in the third stage, the weight loadings of MOFs in 1-NH_2-CuBDC-TAPA-TFP and 5-NH_2-CuBDC-TAPA-TFP MCOFs are respectively ~46% and ~47%. For the 0.25-NH_2-CuBDC-TAPA-TFP and 0.5-NH_2-CuBDC-TAPA-TFP MCOFs, the weight loss occurs in three steps: a) 130-200 °C, only COFs degradation, b) 200-660 °C: MOFs and COFs degradation, c) 660-800 °C: only COFs degradation. The weight loading of MOFs in 0.25-NH_2-CuBDC-TAPA-TFP MCOFs is ~30%.

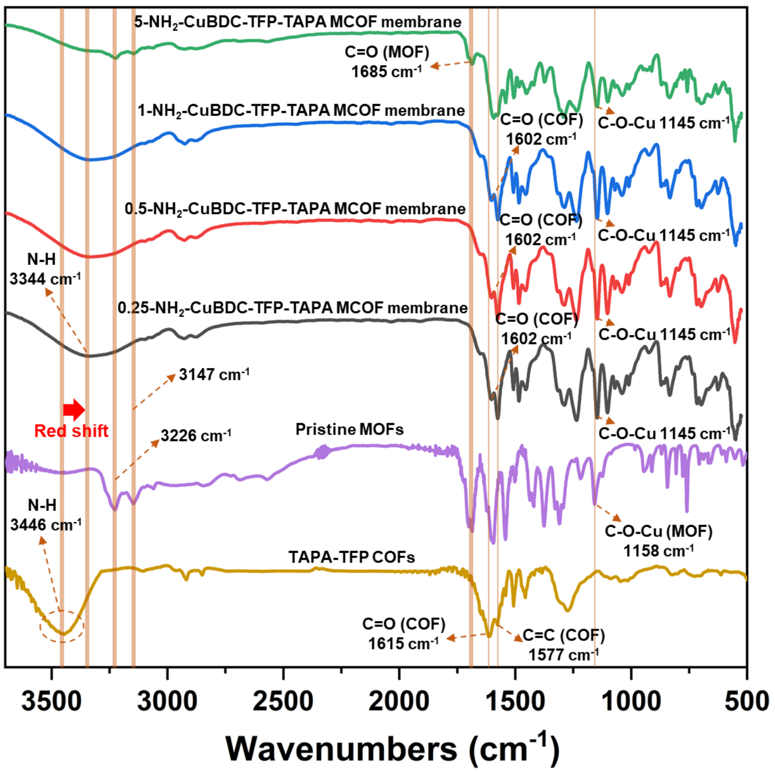

Supplementary Figure 29. ATR-FTIR spectra of synthesized MCOF composite membranes.

Supplementary Notes: The *x*-NH_2-CuBDC-TAPA-TFP MCOFs exhibit nearly consistent spectra. Compared with pristine MOFs, the characteristic peaks from C-O-Cu stretching vibrations shift from 1158 cm^{-1} to 1145 cm^{-1}, demonstrating the change of the MOF coordination environment. Interestingly, for the 5-NH_2-CuBDC-TAPA-TFP MCOFs, the characteristic peak at 1685 cm^{-1} is ascribed to the C=O stretching vibrations of -COOH groups in MOF free ligands and the characteristic peaks at 3226 cm^{-1} and 3147 cm^{-1} are assigned to N-H stretching vibrations of MOFs. Nonetheless, the peaks from C=O and C=C stretching vibrations of COFs are not obvious. Conversely, for the *x*-NH_2-CuBDC-TAPA-TFP MCOFs (*x*=0.25, 0.5, 1), the peaks from C=O and C=C stretching vibrations of COFs can be clearly detected while the characteristic peaks from C=O and N-H stretching vibrations of MOFs are blurry. This indicates that there is a competitive effect between the characteristic peak intensities of MOFs and COFs.

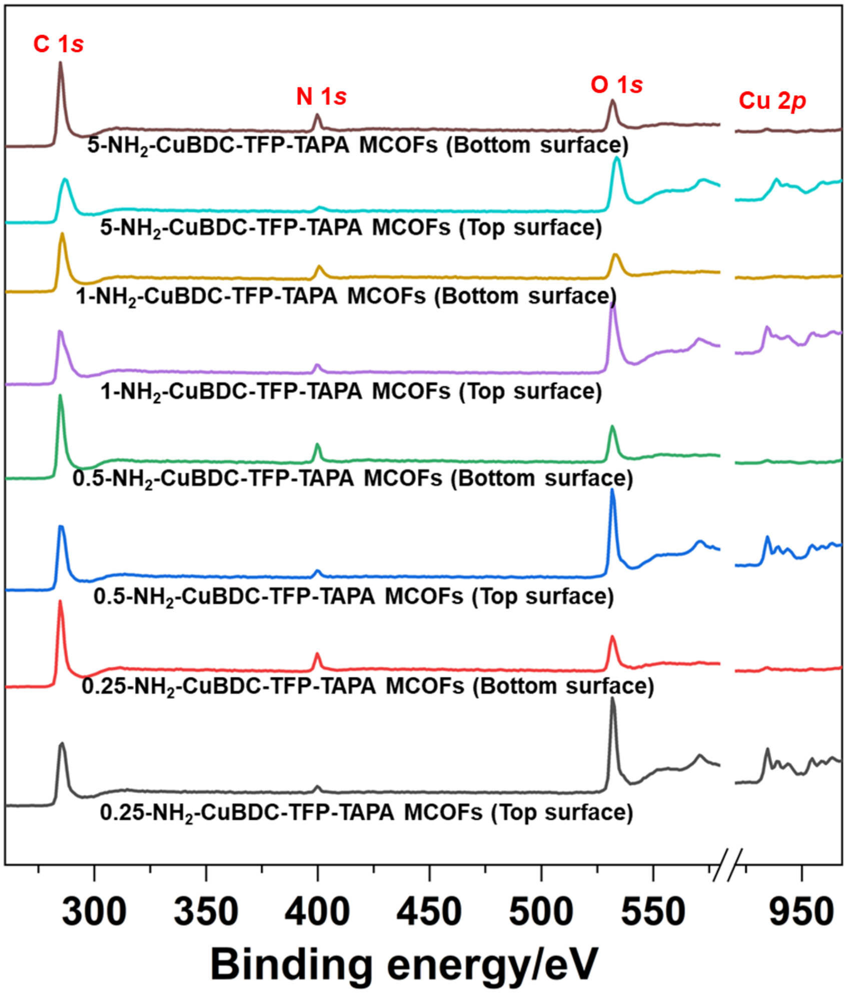

Supplementary Figure 30. XPS spectra of synthesized MCOF composite membranes. The Cu signals confirm the successful introduce of MOFs into MCOFs.

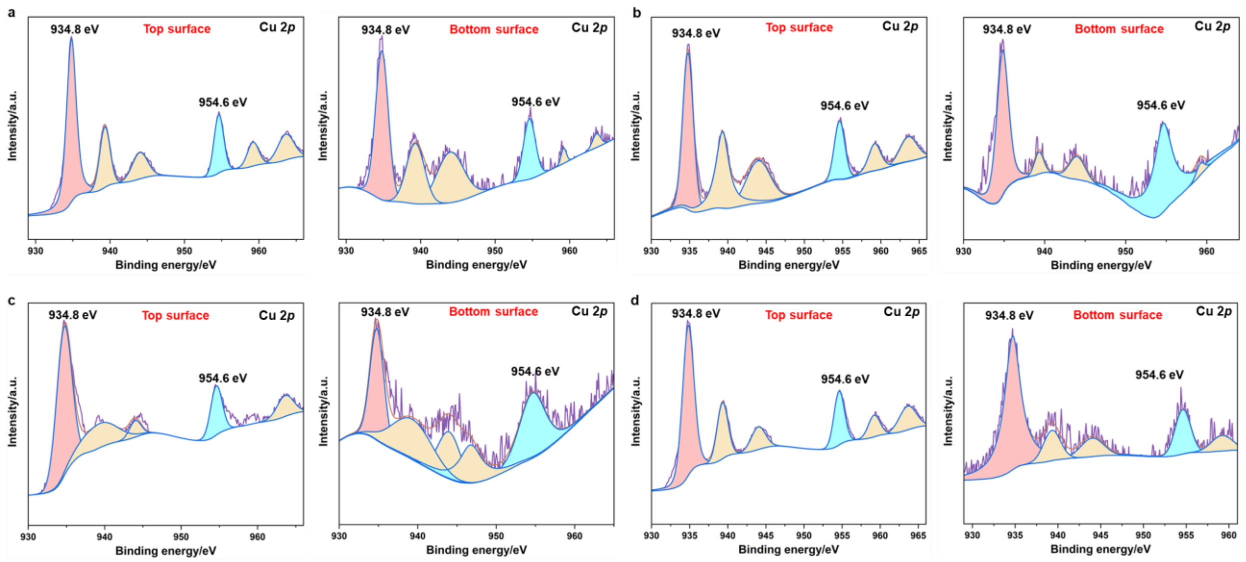

Supplementary Figure 31. Cu 2*p* spectra of 0.25-NH_2-CuBDC-TAPA-TFP (a), 0.5-NH_2-CuBDC-TAPA-TFP (b), 1-NH_2-CuBDC-TAPA-TFP (c) and 5-NH_2-CuBDC-TAPA-TFP (d) MCOF composite membranes.

Supplementary Notes: The binding energy of Cu 2*p* of Cu in MOFs is 935.0 eV and 954.8 eV. It shifts to the slightly lower binding energy of 934.8 eV and 954.6 eV in the MCOF membranes, suggesting the coordination interaction between the -NH groups in COFs and the Cu centers from MOFs. It should be noted that the coordination interaction may be very weak in the MCOF membranes due to the very slight negative shift in the binding energy of Cu 2*p* of Cu (the negative shift of CMOFs is ~0.6 (COFs: 0.5 mM TAPA + 0.5 mM TFP) and ~1.4 eV (COFs: 1 mM TAPA + 1 mM TFP), but the negative shift of MCOFs is ~0.2 eV. The negative shift of CMOFs is 3-fold or 7-fold that of MCOFs.).

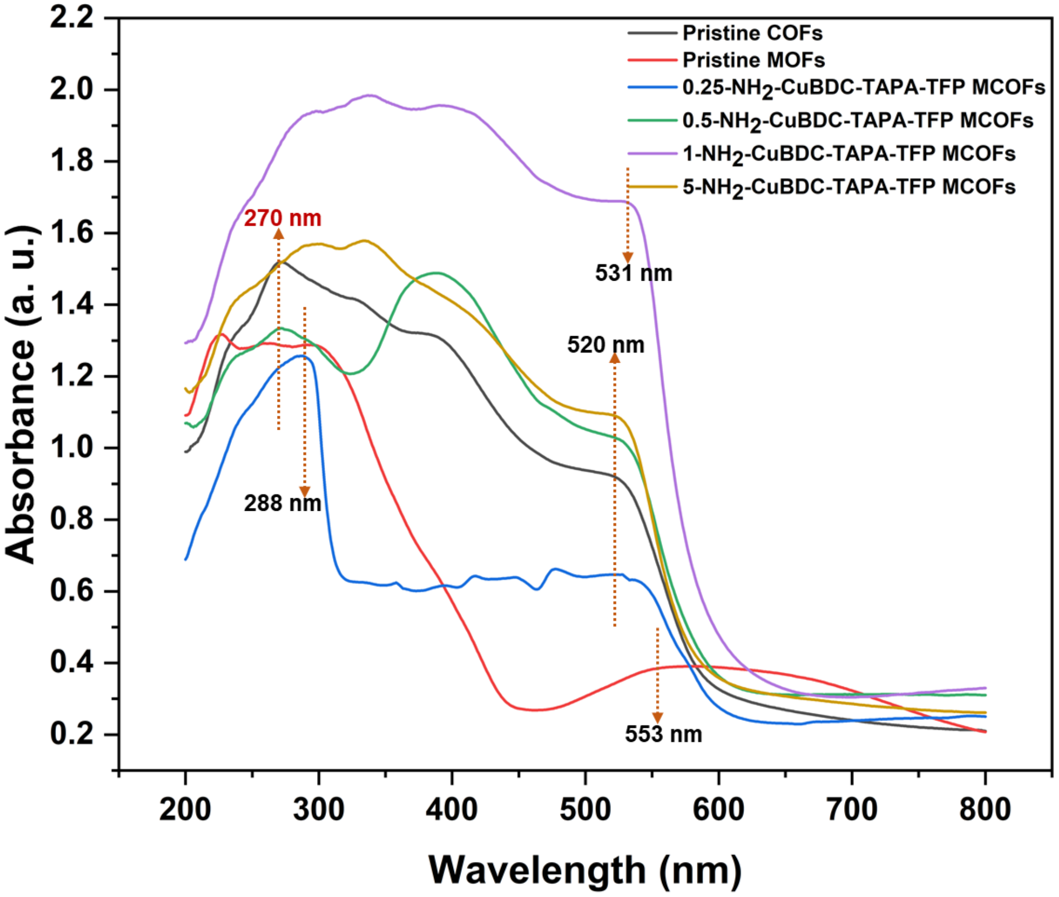

Supplementary Figure 32. UV-Vis-NIR spectra of synthesized MCOF composite membranes.

Supplementary Notes: The pristine COF membrane mainly shows three absorption bands centered at 270 nm, 387 nm and 520 nm. After the introduce of MOFs, different degrees of red-shift are observed in the CMOF composite membranes, which is attributed to the coordination interaction between MOFs and COFs, resulting in a smaller vibration frequency of the chemical bonds. It is worth mentioning that the red-shift is very weak, especially at around 520 nm, indicating that the coordination interaction between MOFs and COFs in MCOF composite membranes is very weak and even negligible.

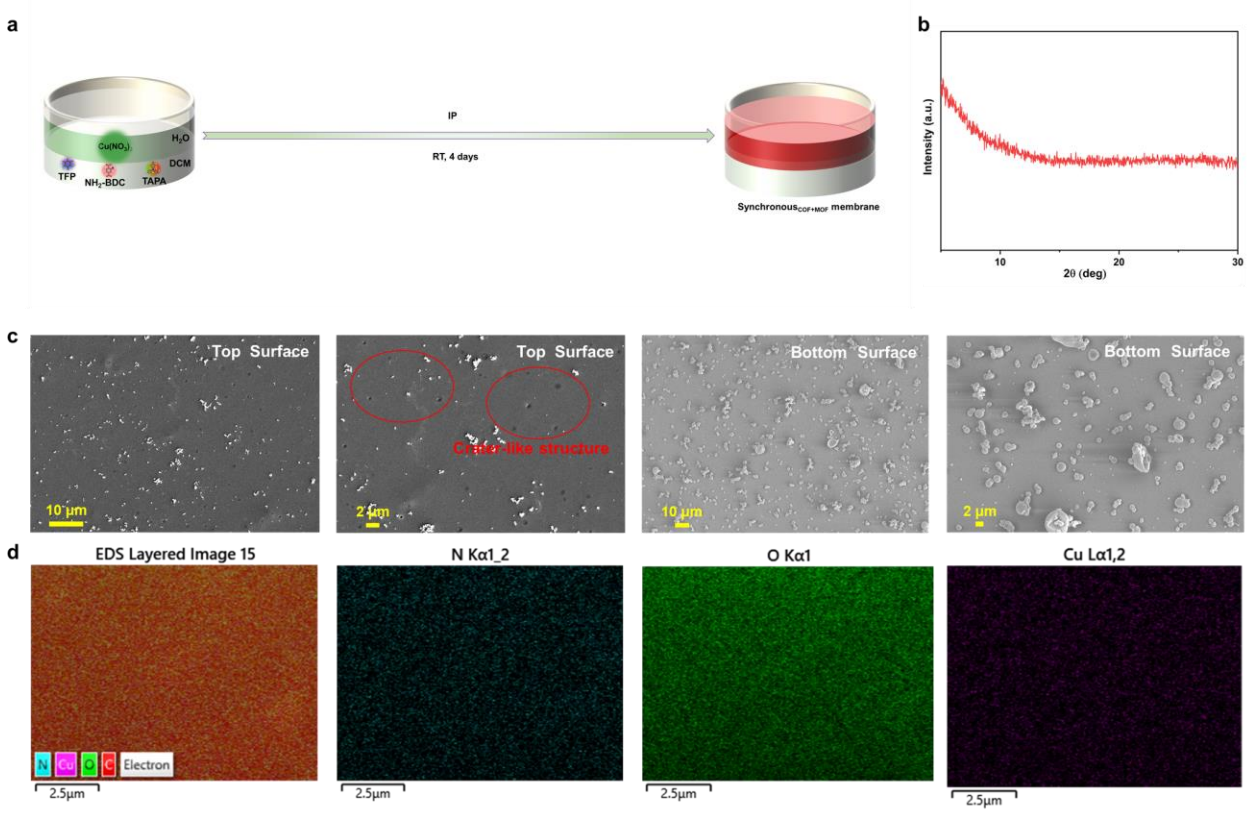

Supplementary Figure 33. Synchronous$_{COF+MOF}$ composite membrane. (a) Schematic illustration of the growth process of the Synchronous$_{COF+MOF}$ composite membrane. (b) XRD profile of the Synchronous$_{COF+MOF}$ composite membrane. (c) SEM images of the top and bottom surfaces of the Synchronous$_{COF+MOF}$ composite membrane. (d) EDXS mapping and elemental distributions of the prepared Synchronous$_{COF+MOF}$ composite membrane. The Cu signals from EDXS confirm a good distribution of MOFs in the composite membrane.

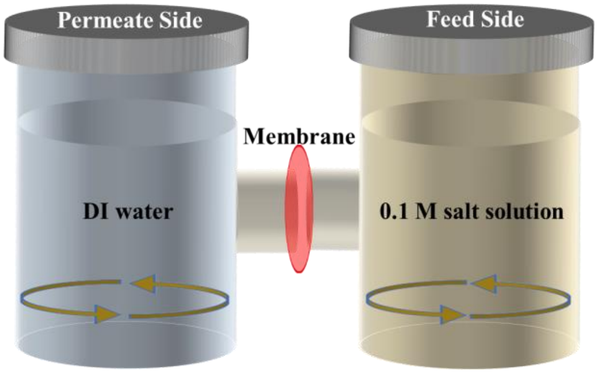

Supplementary Figure 34. Permeation cell employed to investigate the single and binary ion permeation behaviors of as-prepared composite membranes.

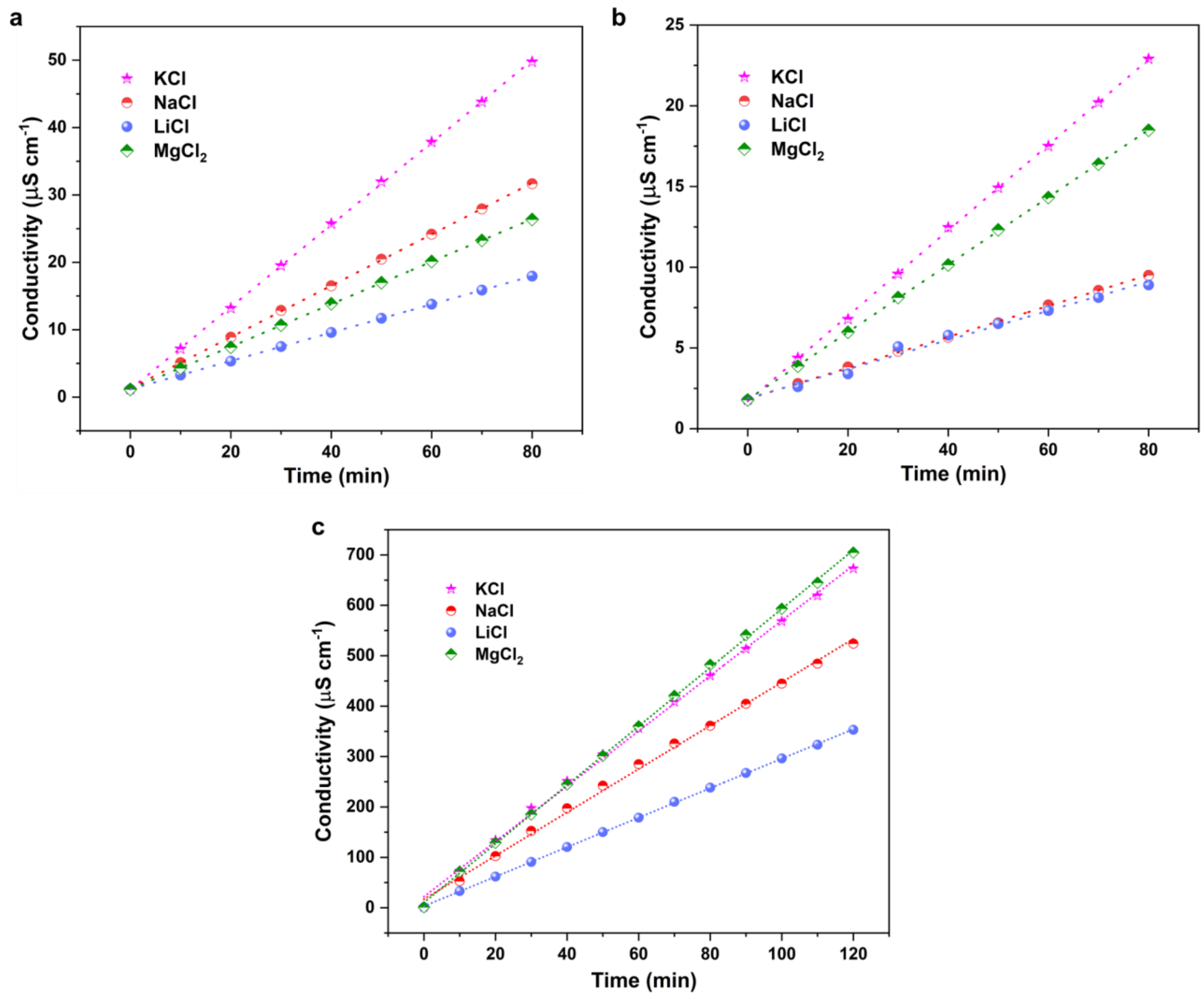

Supplementary Figure 35. Ion conductivity in the permeate side as a function of time for the pristine COF membranes (a, COFs: 0.5 mM TAPA + 0.5 mM TFP. b, COFs: 1 mM TAPA + 1 mM TFP) and PES substrate (c). It should be noted that the pristine COF membrane and PES substrate have no ion selectivity.

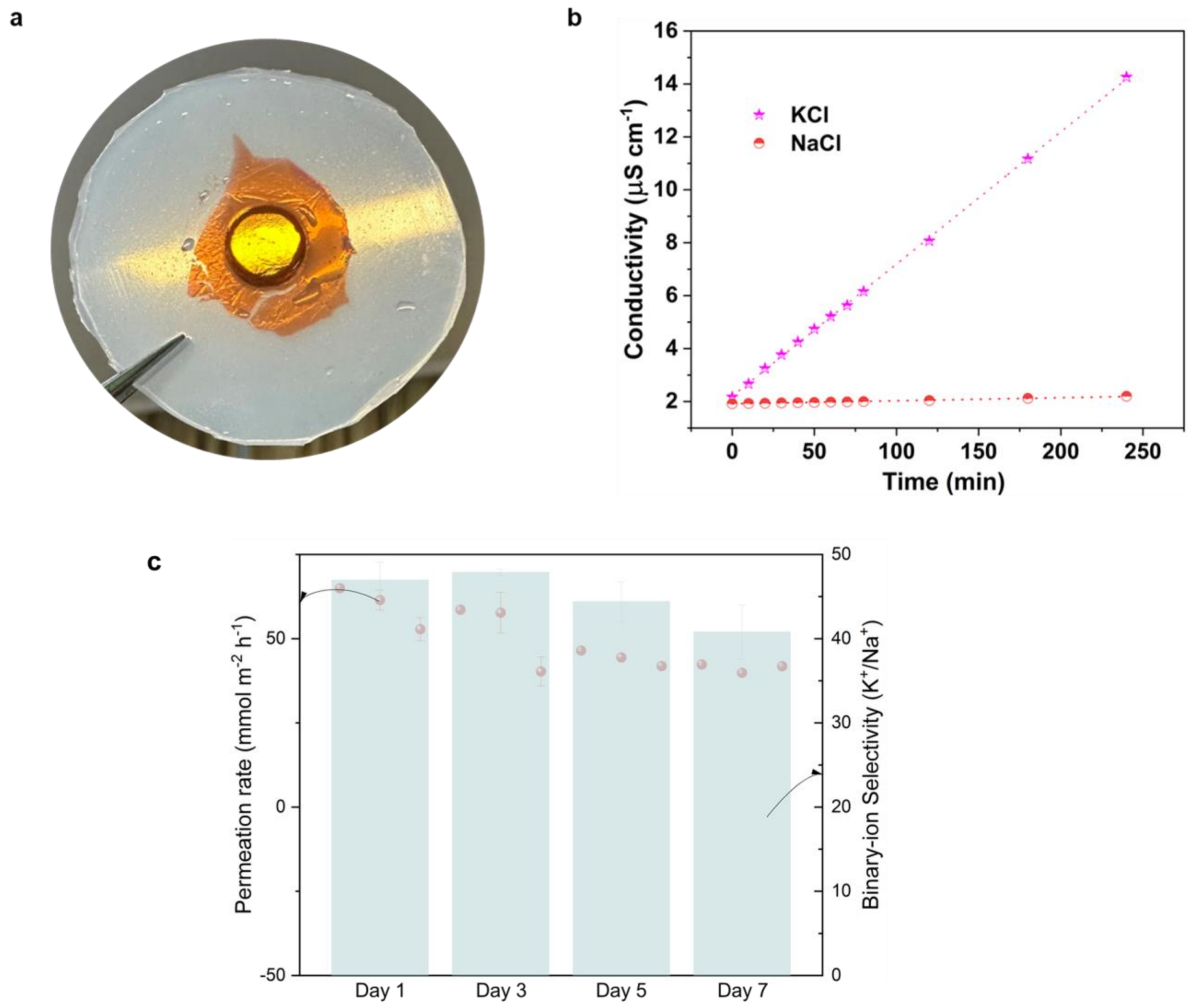

Supplementary Figure 36. (a) TAPA-TFP-0.25-NH_2-CuBDC CMOF composite membranes (COFs: 1 mM TAPA + 1 mM TFP) after 20 hours of testing. (b) The K^+ and Na^+ diffusion behavior in TAPA-TFP-0.25-NH_2-CuBDC CMOF composite membranes (COFs: 1 mM TAPA + 1 mM TFP). (c) Long-term stability test for TAPA-TFP-0.25-NH_2-CuBDC CMOF composite membranes (COFs: 1 mM TAPA + 1 mM TFP).

Supplementary Notes: For **Supplementary Figure 36c**, the COF membranes were tested for 7 hours every day, with samples being taken and tested at the 1st, 3rd, and 7th hours respectively. Then the COF membranes were washed, and immersed in pure water and left to stand for the test on the next day.

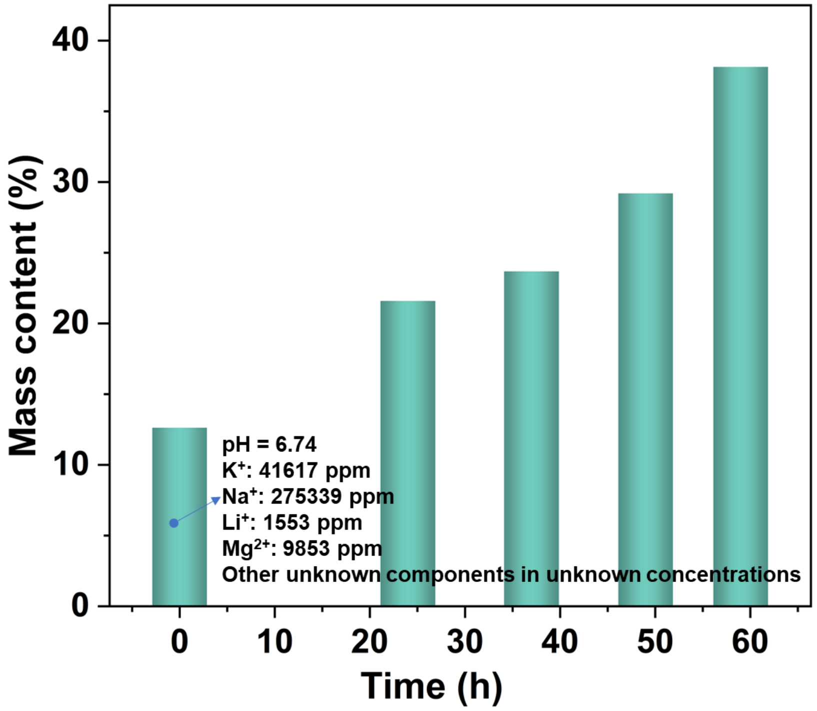

Supplementary Figure 37. K^+ mass content as a function of penetration duration when employing practical brine from Sichuan deep ground as the separation system.

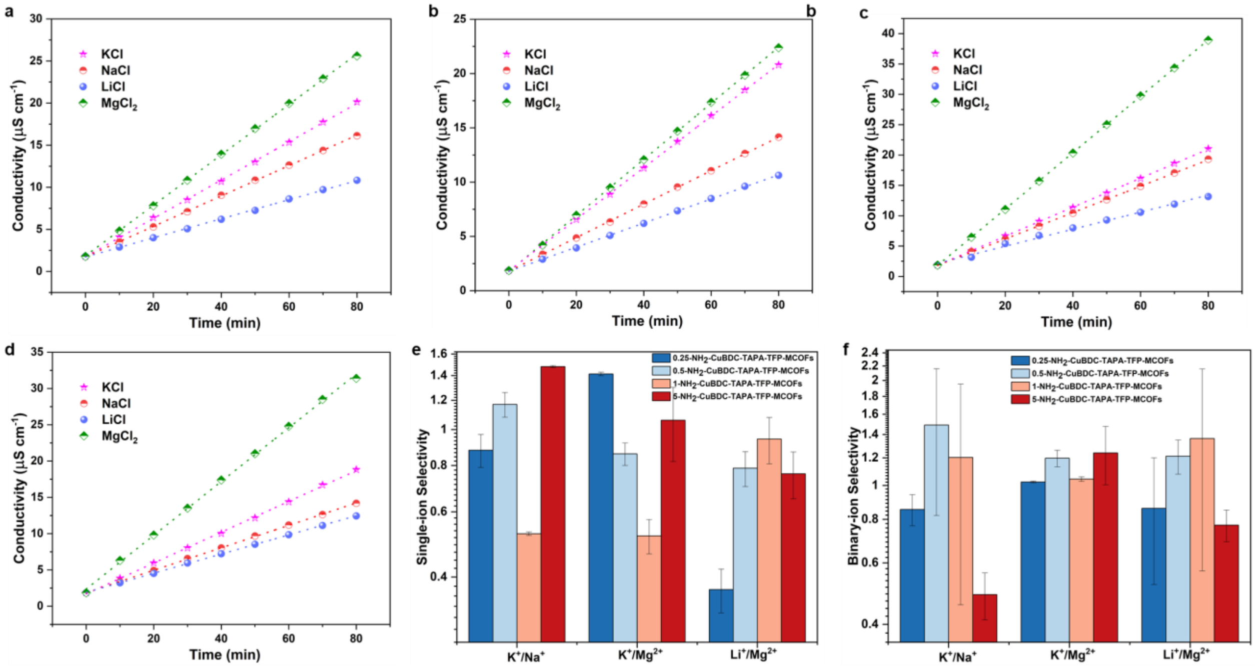

Supplementary Figure 38. Ion conductivity in the permeate side as a function of time for the 0.25-NH_2-CuBDC-TAPA-TFP (a), 0.5-NH_2-CuBDC-TAPA-TFP (b), 1-NH_2-CuBDC-TAPA-TFP (c) and 5-NH_2-CuBDC-TAPA-TFP (d) MCOF composite membranes. Single-ion (e) and binary-ion (f) selectivity of the MCOF composite membranes.

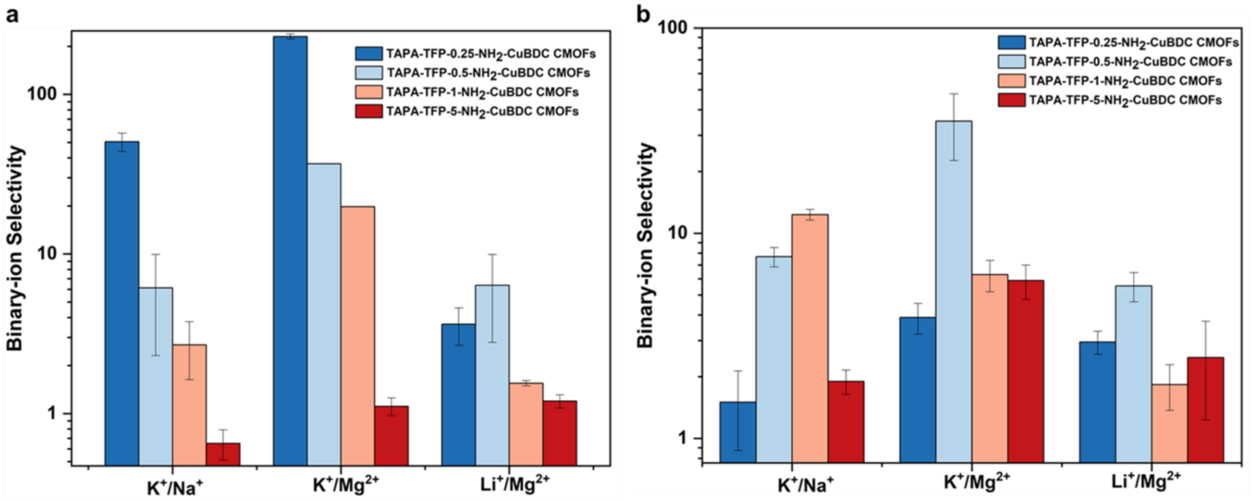

Supplementary Figure 39. Binary-ion selectivity of the CMOF composite membranes. a. COFs: 1 mM TAPA + 1 mM TFP. b. COFs: 0.5 mM TAPA + 0.5 mM TFP.

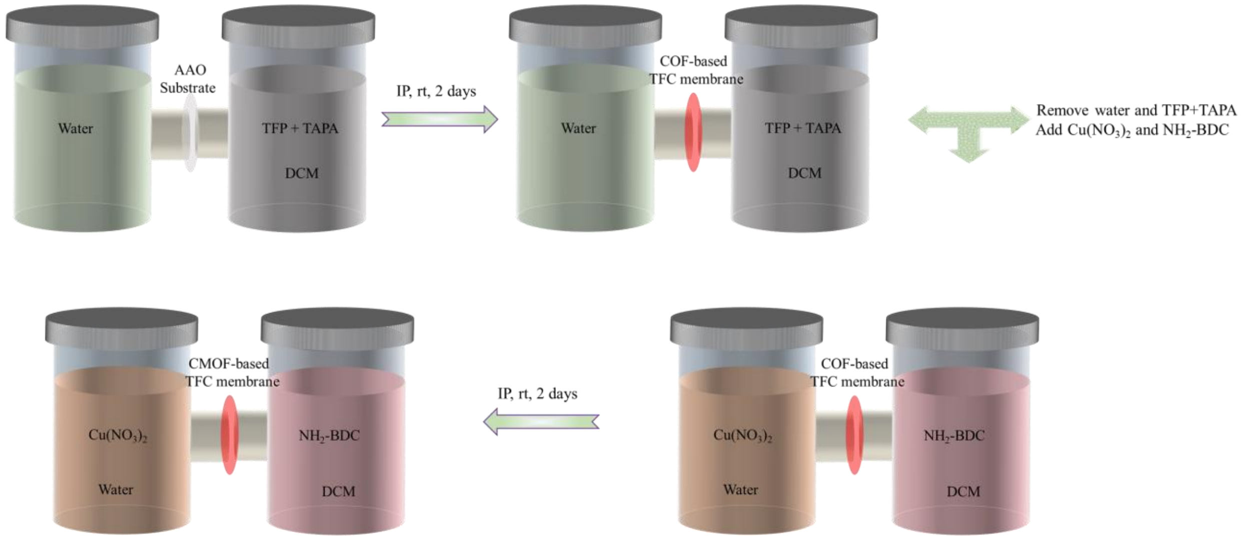

Supplementary Figure 40. Schematic illustration of the fabrication process of CMOF-based TFC membranes on AAO substrates.

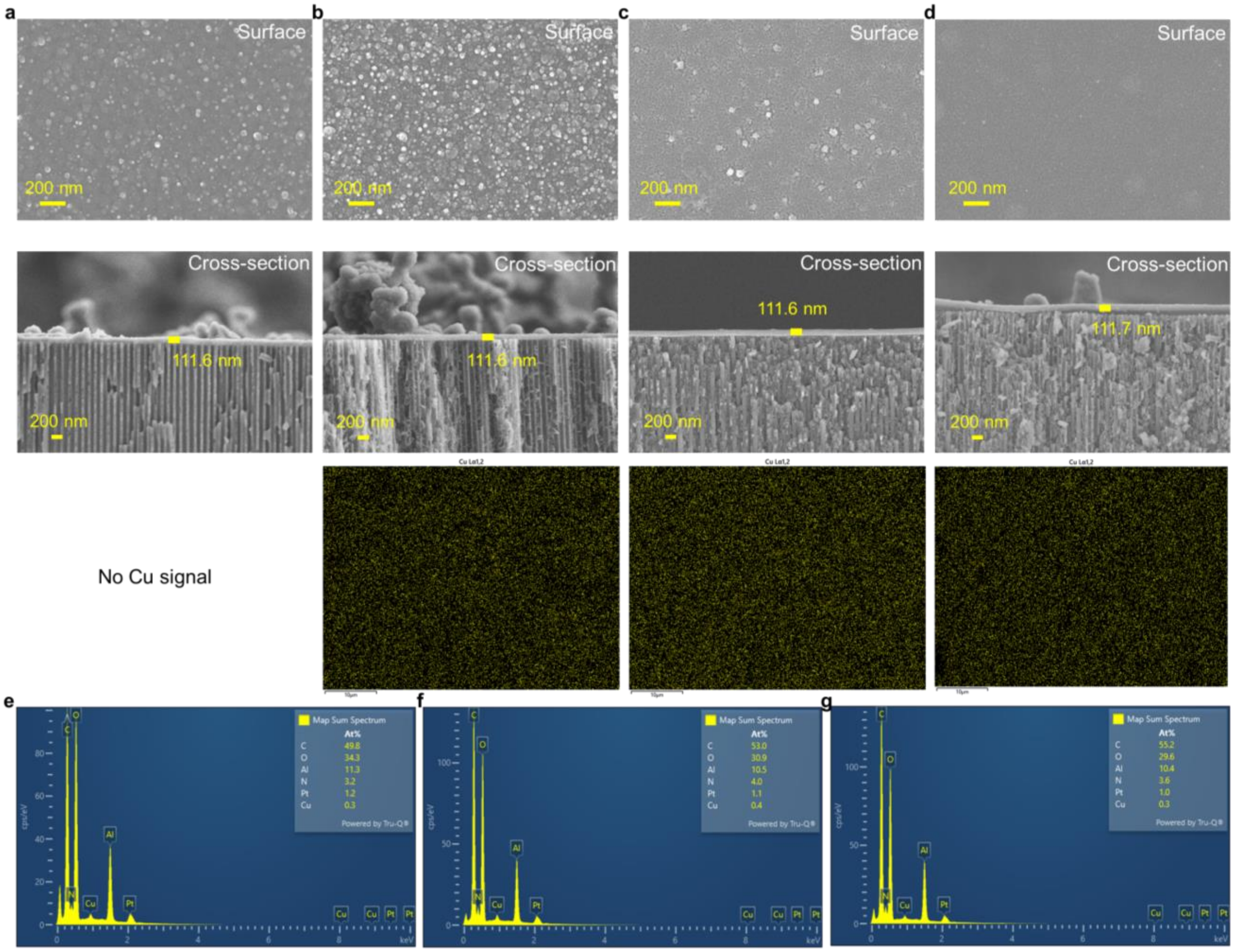

Supplementary Figure 41. SEM images and EDXS mapping of pristine TAPA-TFP COF (COFs: 2 mM TAPA + 2 mM TFP) (**a**), TAPA-TFP-0.25-NH_2-CuBDC CMOF (**b**), TAPA-TFP-0.5-NH_2-CuBDC CMOF (**c**), and TAPA-TFP-1-NH_2-CuBDC CMOF (**d**) -based TFC membranes. Corresponding atomic compositions of TAPA-TFP-0.25-NH_2-CuBDC CMOF (**e**), TAPA-TFP-0.5-NH_2-CuBDC CMOF (**f**), and TAPA-TFP-1-NH_2-CuBDC CMOF (**g**) -based TFC membranes obtained from EDXS mapping.

Supplementary Notes: Obviously, when grown on the AAO substrate, the pristine COF membrane has a smaller thickness of 111.6 nm compared with pristine self-standing COF membrane grown on the oil-water interface (457.8 nm, Supplementary Figure 1). Moreover, after the introduction of MOFs, the thickness of the TFC membrane hardly changes at all compared to

the pristine COF membrane, which is completely different from the situation in the self-standing membranes. It is attributed to the different synthesis methods and membrane growth environment. Moremore, with the increase of MOF ligand concentration, the surface of the TFC membrane becomes smoother, but we fail to find definite MOFs. Scattered pieces of MOFs can be found in the AAO channels.

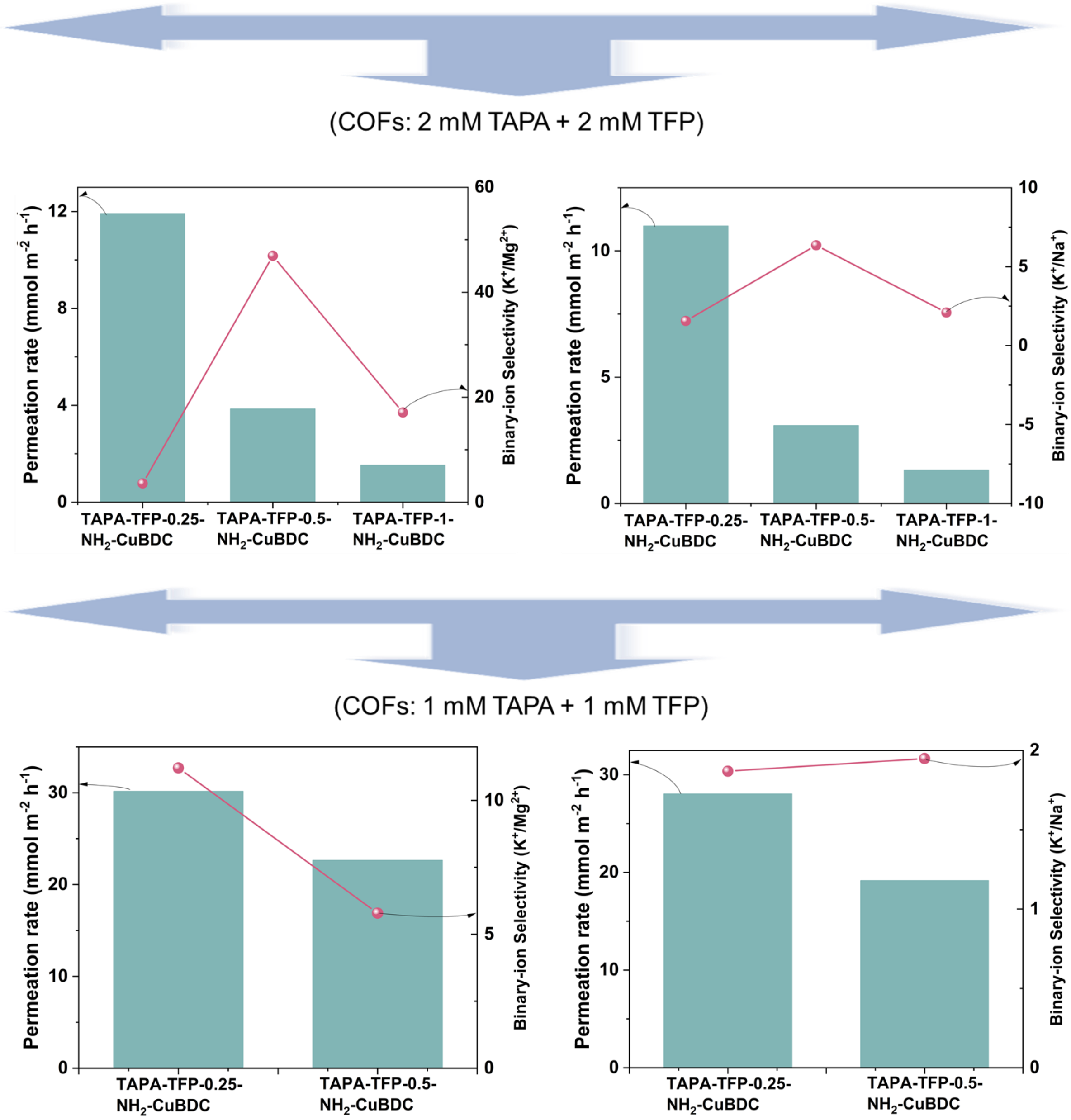

Supplementary Figure 42. Binary-ion selectivity of the prepared TFC membranes.

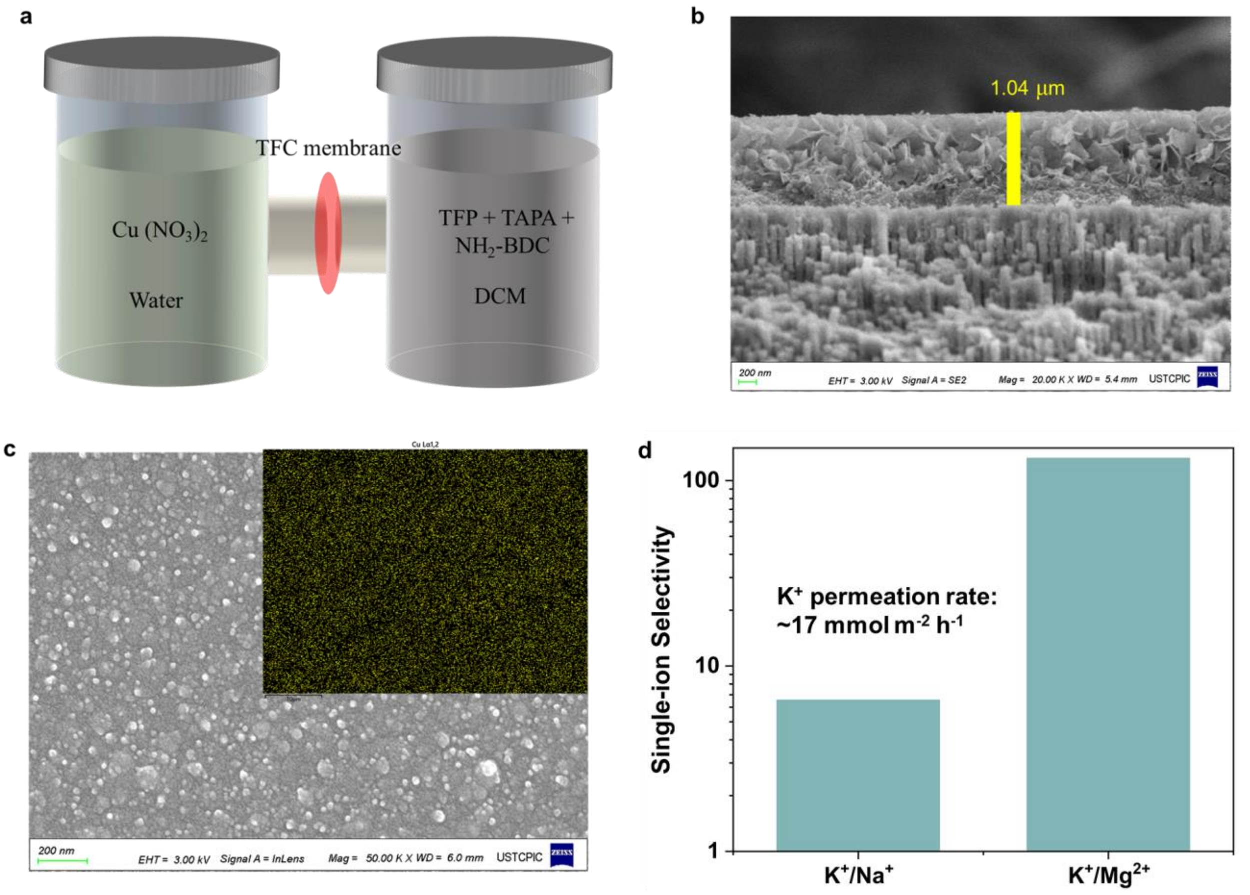

Supplementary Figure 43. Schematic illustration of the fabrication process of the TFC membrane on AAO substrates (a). SEM image of the cross-section of the TFC membrane (b). SEM image and EDXS mapping (inset) of the top surface of the TFC membrane (c). Ion selectivity of the prepared TFC membrane (d).

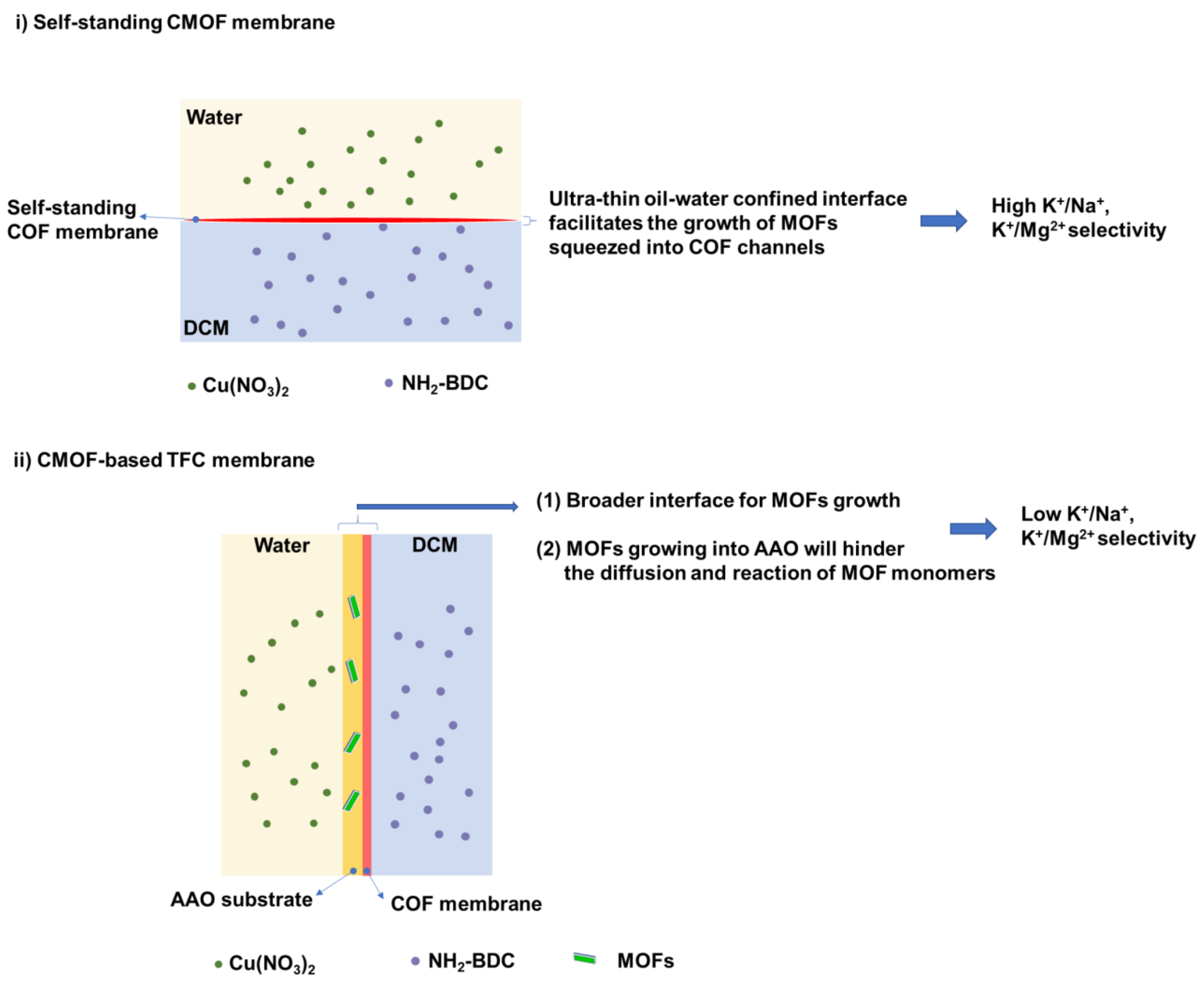

Supplementary Figure 44. Schematic illustration of the different growth environment of CMOF-based TFC membranes and self-standing CMOF membranes.

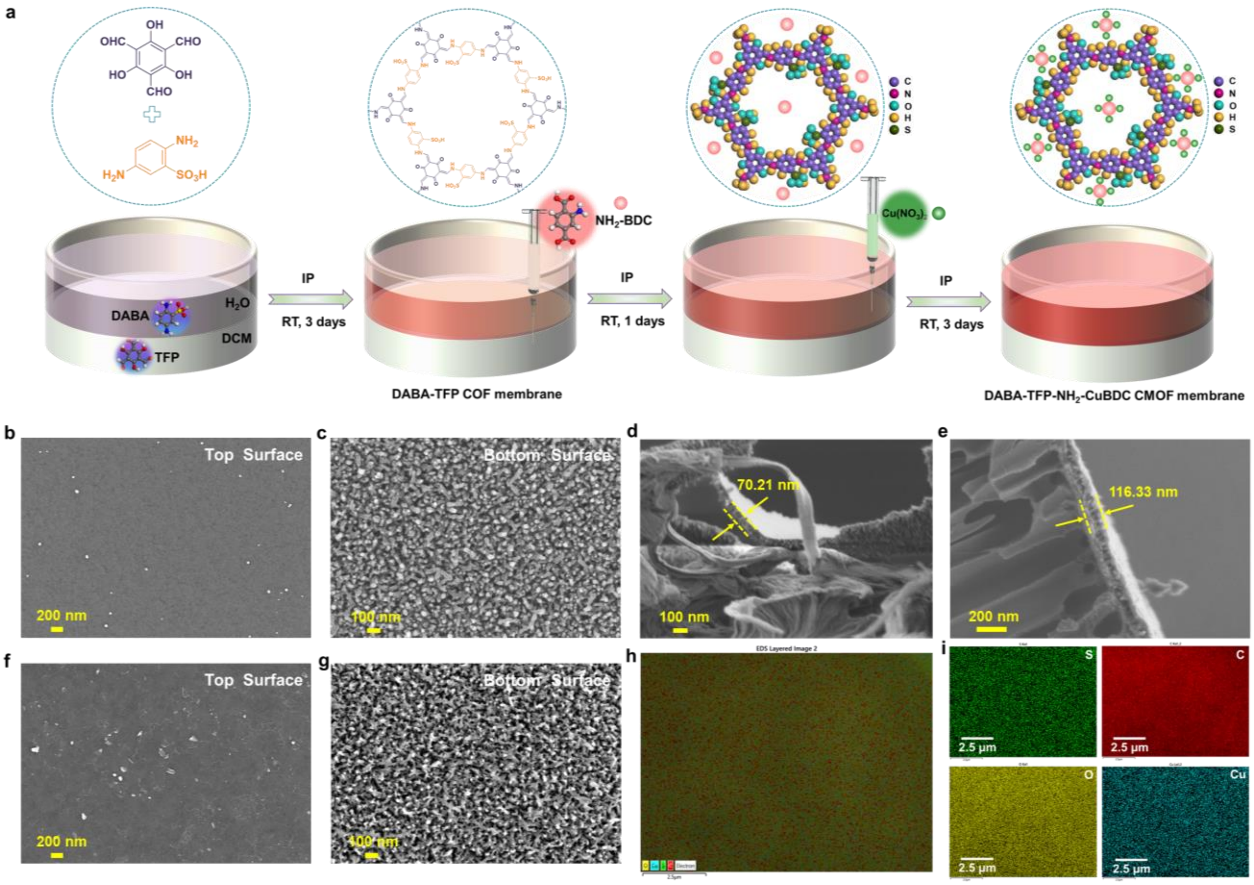

Supplementary Figure 45. DABA-TFP-NH$_2$-CuBDC CMOF composite membrane. (a) Schematic illustration of the growth process of DABA-TFP-NH$_2$-CuBDC CMOF composite membrane. SEM images of the top surface (b), bottom surface (c), and cross-section (d) of the pristine DABA-TFP COF membrane. SEM images of the top surface (f), bottom surface (g), and cross-section (e) of the DABA-TFP-NH$_2$-CuBDC CMOF composite membrane. (h) EDXS mapping and elemental distributions of prepared composite membranes, and the Cu signals from EDXS confirm a good distribution of MOFs in the composite membrane (i).

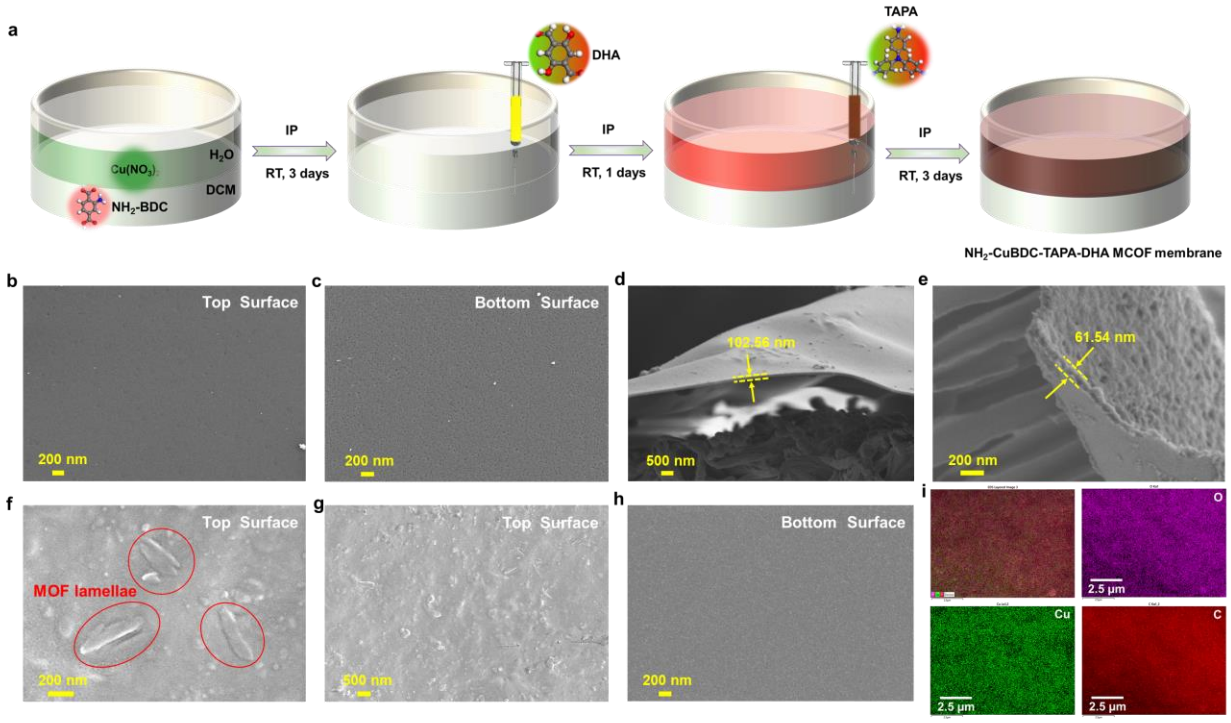

Supplementary Figure 46. NH_2-CuBDC-TAPA-DHA MCOF composite membrane. (a) Schematic illustration of the growth process of NH_2-CuBDC-TAPA-DHA MCOF composite membrane. SEM images of the top surface (b), bottom surface (c), and cross-section (d) of the pristine TAPA-DHA COF membrane. SEM images of the top surface (f and g show different magnification levels.), bottom surface (h), and cross-section (e) of the NH_2-CuBDC-TAPA-DHA MCOF composite membrane. (i) EDXS mapping and elemental distributions of prepared composite membranes. The Cu signals from EDXS confirm a good distribution of MOFs in the composite membrane.

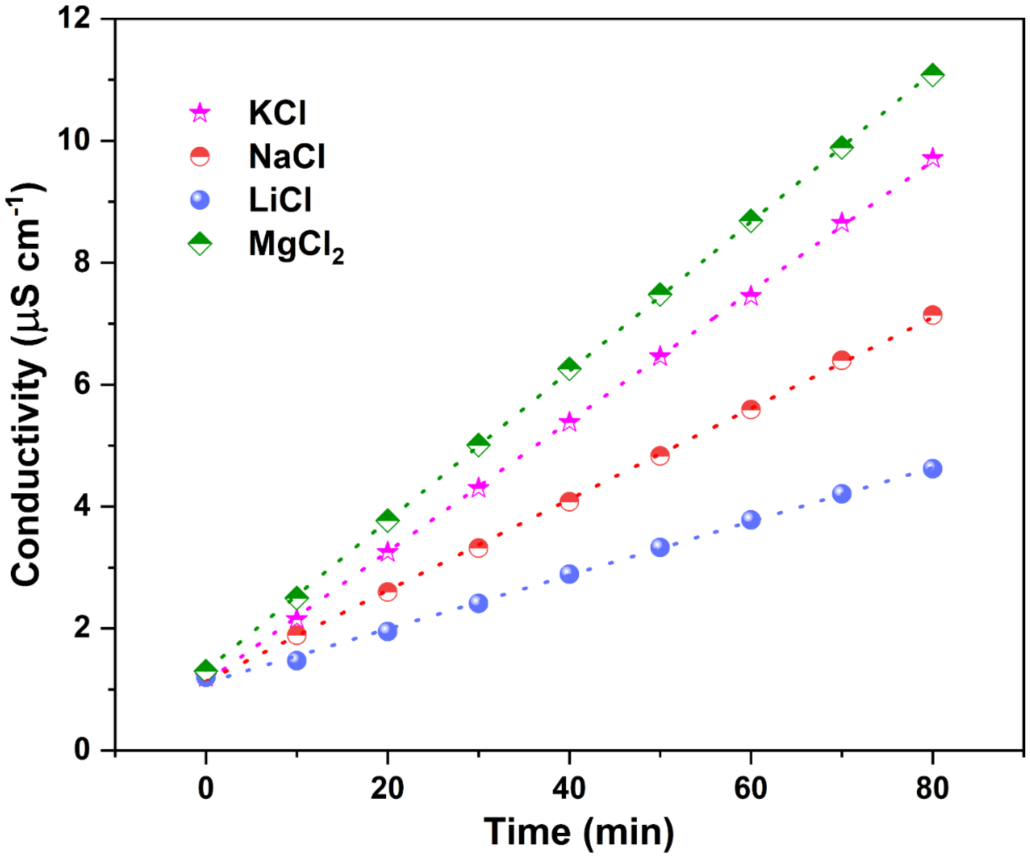

Supplementary Figure 47. Ion conductivity in the permeate side as a function of time for the NH_2-CuBDC-TAPA-DHA MCOF composite membrane.

Supplementary Table 1. Mulliken atom charges of TAPA-TFP COFs in vacuum.

Atom	Charge	Atom	Charge	Atom	Charge	Atom	Charge
1 C	-0.09663	31 H	0.088166	61 C	-0.10396	91 C	0.255127
2 C	-0.08515	32 H	0.087624	62 C	-0.09678	92 C	-0.11012
3 C	-0.09671	33 H	0.100307	63 C	-0.08516	93 C	-0.14125
4 C	-0.104	34 H	0.099277	64 C	-0.09665	94 C	0.332992
5 C	0.246484	35 H	0.150322	65 C	-0.10483	95 C	-0.11165
6 C	-0.10509	36 H	0.103687	66 N	-0.62558	96 C	-0.12277
7 C	-0.07475	37 H	0.102306	67 N	-0.64204	97 C	0.245859
8 C	0.376887	38 H	0.107459	68 H	0.088174	98 C	-0.10376
9 C	-0.07494	39 H	0.087604	69 H	0.087624	99 C	-0.0968
10 C	0.376907	40 H	0.088089	70 H	0.100316	100 C	-0.08513
11 C	-0.07494	41 H	0.099331	71 H	0.099288	101 C	-0.09667
12 C	0.376827	42 H	0.317747	72 H	0.150301	102 C	-0.10476
13 C	0.161324	43 H	0.083395	73 H	0.10369	103 N	-0.62551
14 C	0.254833	44 H	0.08344	74 H	0.10229	104 N	-0.64201
15 C	-0.11001	45 H	0.100307	75 H	0.107451	105 H	0.088165
16 C	-0.14123	46 H	0.106853	76 H	0.087615	106 H	0.087617
17 C	0.333057	47 C	-0.09664	77 H	0.088097	107 H	0.100327
18 C	-0.11166	48 C	-0.08514	78 H	0.099298	108 H	0.099294
19 C	-0.12267	49 C	-0.09672	79 H	0.317794	109 H	0.150327
20 C	0.246318	50 C	-0.10394	80 H	0.083403	110 H	0.103669
21 C	-0.10391	51 C	0.246429	81 H	0.083447	111 H	0.102307
22 C	-0.09676	52 C	-0.10512	82 H	0.10035	112 H	0.107489
23 C	-0.08518	53 C	0.161327	83 H	0.106842	113 H	0.087646
24 C	-0.09666	54 C	0.254997	84 C	-0.09663	114 H	0.088124
25 C	-0.10499	55 C	-0.11006	85 C	-0.08517	115 H	0.099334
26 N	-0.6256	56 C	-0.14128	86 C	-0.09671	116 H	0.317655
27 N	-0.64206	57 C	0.333047	87 C	-0.10405	117 H	0.083433
28 O	-0.62171	58 C	-0.11162	88 C	0.246585	118 H	0.083432
29 O	-0.62171	59 C	-0.12271	89 C	-0.10517	119 H	0.100344
30 O	-0.62158	60 C	0.246172	90 C	0.161215	120 H	0.106844

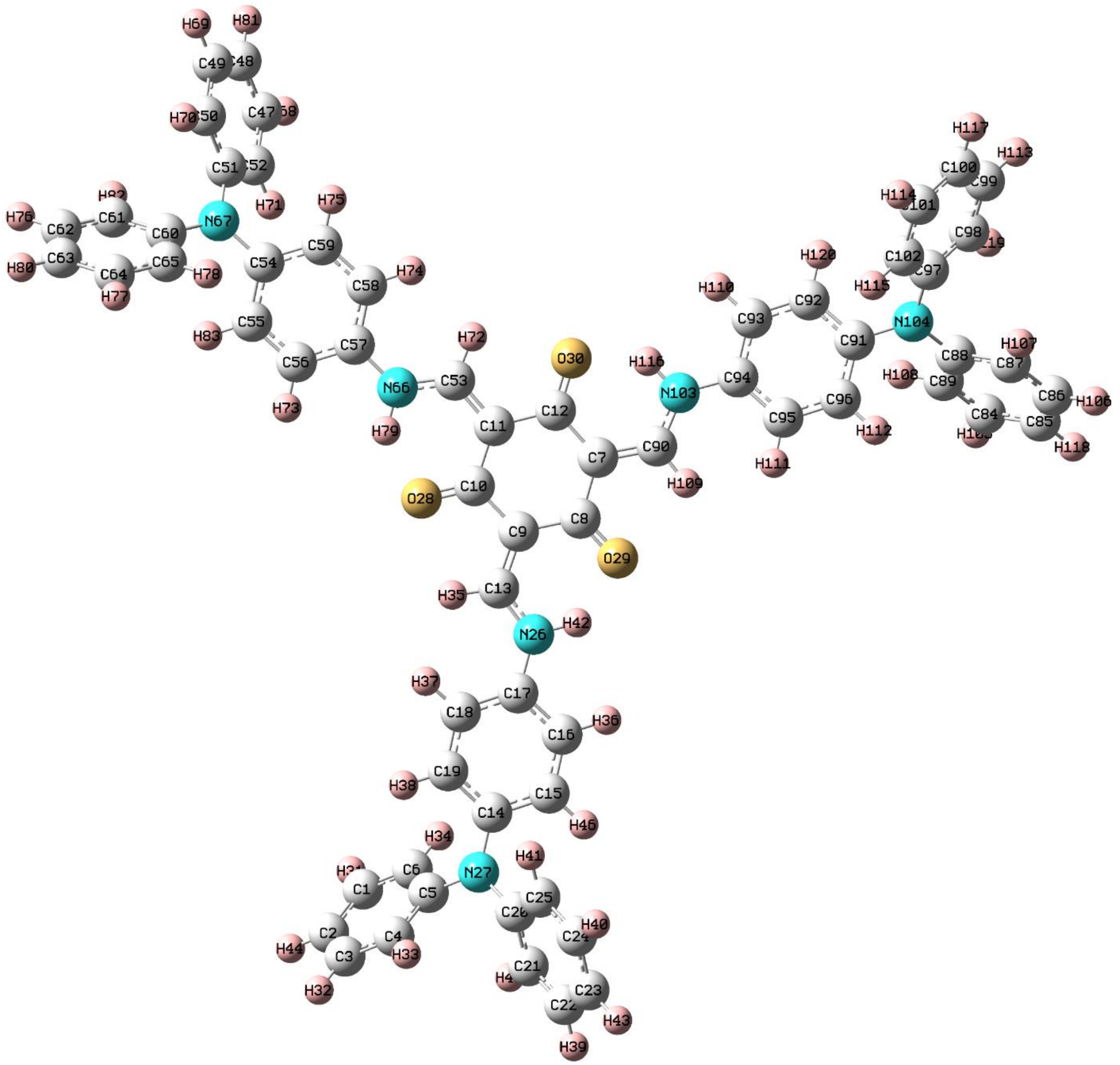
H69
H81
C49
C48
H70
C47
C51
C52
H76
C61
C60
N67
H71
H75
C62
C63
H80
C64
C65
H78
H77
C54
C59
H74
C58
C55
H83
C57
C56
H72
C53
N66
H73
H79
O30
C12
C11
C7
C10
O28
C9
C8
O29
C90
H109
H116
N103
C94
C95
H111
C96
H112
H110
C93
C92
H120
C91
N104
C97
C102
H115
H114
C98
C100
C99
H117
H113
C88
C87
H107
H108
C89
C84
C85
C86
H106
H118
H35
C13
N26
H42
H37
C17
C18
C16
H36
C19
H38
C15
C14
H46
H34
C6
C1
C5
N27
H41
C25
H40
C24
C2
C4
H44
C3
H33
H32
C21
C23
C22
H43
H39

Supplementary Table 2. Atomic composition of synthesized TAPA-TFP-*x*-NH_2-CuBDC CMOF composite membranes from XPS spectra (COFs: 0.5 mM TAPA + 0.5 mM TFP).

Sample		Atomic composition (%)			
		C	N	O	Cu
**TAPA-TFP COFs**	Top surface	71.3	8.3	20.4	/
	Bottom surface	72.8	8.3	18.9	/
**TAPA-TFP-0.25-NH_2-CuBDC CMOFs**	Top surface	75.2	8.4	15.7	0.7
	Bottom surface	70.7	7.2	21.0	1.1
**TAPA-TFP-0.5-NH_2-CuBDC CMOFs**	Top surface	72.6	6.8	20.0	0.6
	Bottom surface	72.6	7.3	19.0	1.1
**TAPA-TFP-1-NH_2-CuBDC CMOFs**	Top surface	73.3	7.5	18.3	0.9
	Bottom surface	72.9	6.7	19.1	1.3
**TAPA-TFP-5-NH_2-CuBDC CMOFs**	Top surface	71.8	8.2	18.9	1.1
	Bottom surface	74.9	7.8	16.1	1.2
**Pristine MOFs**	/	64.0	7.1	25.7	3.3

Supplementary Notes: Cu element is detected on the top and bottom surfaces of TAPA-TFP-*x*-NH_2-CuBDC CMOF membrane and becomes increasingly abundant with increasing ligand concentration.

Supplementary Table 3. Atomic composition of synthesized TAPA-TFP-*x*-NH_2-CuBDC CMOF composite membranes from XPS spectra (COFs: 1 mM TAPA + 1 mM TFP).

**Sample**		**Atomic composition (%)**			
		**C**	**N**	**O**	**Cu**
**TAPA-TFP COFs**	Top surface	69.9	7.6	22.5	/
	Bottom surface	71.2	7.7	21.1	/
**TAPA-TFP-0.25-NH_2-CuBDC CMOFs**	Top surface	72.5	6.1	20.5	0.9
	Bottom surface	73.9	7.0	18.1	1.0
**TAPA-TFP-0.5-NH_2-CuBDC CMOFs**	Top surface	72.4	5.6	21.0	1.0
	Bottom surface	73.5	5.8	19.5	1.2
**TAPA-TFP-1-NH_2-CuBDC CMOFs**	Top surface	73.8	5.7	19.8	0.7
	Bottom surface	75.0	5.5	18.5	1.0
**TAPA-TFP-5-NH_2-CuBDC CMOFs**	Top surface	72.1	6.2	20.7	1.0
	Bottom surface	75.8	6.4	16.8	1.1

Supplementary Notes: It is worth noting that when the thickness of pristine COF membrane is increased, the content of MOFs introduced does not change much, indicating that the inclusion capacity of COF membrane to MOFs is finite.

Supplementary Table 4. Atomic composition of synthesized *x*-NH_2-CuBDC-TAPA-TFP MCOF composite membranes from XPS spectra.

**Sample**		**Atomic composition (%)**			
		**C**	**N**	**O**	**Cu**
**0.25-NH_2-CuBDC-TFP-TAPA MCOFs**	Top surface	66.1	2.6	26.9	4.3
	Bottom surface	78.8	7.8	12.9	0.5
**0.5-NH_2-CuBDC-TFP-TAPA MCOFs**	Top surface	68.2	3.5	24.6	3.7
	Bottom surface	78	7.9	13.7	0.4
**1-NH_2-CuBDC-TFP-TAPA MCOFs**	Top surface	63.8	5.0	27	4.2
	Bottom surface	76.6	8.7	14.1	0.5
**5-NH_2-CuBDC-TFP-TAPA MCOFs**	Top surface	68.4	3.1	26	2.5
	Bottom surface	79.4	7.7	12.6	0.3

Supplementary Table 5. Properties of cations used in transport experiments.

Ion	Bare diameter (Å)	Hydrated diameter (Å)	Hydrated energy (kJ mol^{-1})
K^+	2.66	6.62	-295
Na^+	1.90	7.16	-365
Li^+	1.20	7.64	-475
Mg^{2+}	1.30	8.56	-1830

Supplementary Table 6. Comparison of the ion permeation rate and ion selectivity of different membranes reported in the literature.

Membrane	Permeation rate (K^+, Na^+) (mmol m^{-2} h^{-1})	Ideal Ion selectivity (K^+/Na^+)	Actual Ion selectivity (K^+/Na^+)	Concentration (M)	Ref.
**$TpBDMe_2$ membranes**	195, 174	1.12	1.64	0.1	[26]
**$TpPa-SO_3H$ membrane**	45, 4.5	10	2.5	0.1	[28]
**$TpPa-PO_3H_2$ membrane**	67, 6	11.17	4.1	0.1	[28]
**$TpPa-CO_2H$ membrane**	29, 23	1.26	1.1	0.1	[28]
**CC3 membranes**	2062, 1322	1.56	1.34	0.1	[27]
**CPOS pores**	94.4, 5.2	18.15	/	0.1	[29]
**COF-Cys-60% membrane**	16.2, 9.1	1.8	/	0.1	[16]
**MLM-EDTA-1.5**	88, 37	2.38	/	0.2	[30]
**PCGO membranes**	6.5, 4.2	1.55	/	1	[31]
**EtOH-M**	25.4, 15.8	1.61	/	0.2	[32]
**24 h-FRGO membrane**	182, 74	2.46	/	0.1	[33]
**ACN-3**	1.4, 3	0.47	/	0.2	[34]
**LDH-M**	4200, 2800	1.5	/	1	[35]
**$COF-AzoSO_3H$ membrane**	330, 250	1.32	/	0.1	[36]
**TbDa-Azo membrane**	740, 620	1.19	/	0.2	[37]
**3D-COOH-COF membrane**	1500, 1200	1.25	/	0.1	[38]
**TpHZ-T membrane**	7.23, 23.6	0.31	/	0.1	[39]
**TpHZ-D membrane**	3.76, 19.68	0.19	/	0.1	[39]
**$TAPA-TFP-0.5-NH_2-CuBDC$ CMOF***	125.46, 9.39	14.69	7.69	0.1	This work
**$TAPA-TFP-0.25-NH_2-CuBDC$ CMOF****	49.38, 0.6	82.52	50.47	0.1	This work

* Pristine COFs: 0.5 mM TAPA + 0.5 mM TFP. ** Pristine COFs: 1 mM TAPA + 1 mM TFP.

Supplementary References

1. Y. Liu, H. Wu, R. Li, J. Wang, Y. Kong, Z. Guo, H. Jiang, Y. Ren, Y. Pu, X. Liang, F. Pan, Y. Cao, S. Song, G. He, Z. Jiang. MOF-COF “alloy” membranes for efficient propylene/propane separation. *Adv. Mater*. **16**, 2201423 (2022).

2. W. Sun, X. Tang, Q. Yang, Y. Xu, F. Wu, S. Guo, Y. Zhang, M. Wu, Y. Wang. Coordination-induced interlinked covalent- and metal–organic-framework hybrids for enhanced lithium storage. *Adv. Mater*. **31**, 1903176 (2019).

3. H. Fan, M. Peng, I. Strauss, A. Mundstock, H. Meng, J. Caro. MOF-in-COF molecular sieving membrane for selective hydrogen separation. *Nat. Commun*. **12**, 38 (2021).

4. L. Cao, I-C. Chen, Z. Li, X. Liu, M. Mubashir, R. A. Nuaimi, Z. Lai. Switchable Na^+ and K^+ selectivity in an amino acid functionalized 2D covalent organic framework membrane. *Nat. Commun*. **13**, 7894 (2022).
