# Supplementary material for: Biomimetic KcsA channels enabled by 1D MOF-in-2D COF
Source: Nat Commun. 2025 Oct 14;16:9099. doi: 10.1038/s41467-025-63265-w (PMC12521740; doi:10.1038/s41467-025-63265-w)
Supplement: Supplementary file 2 — Description of Additional Supplementary Files [file 41467_2025_63265_MOESM2_ESM.pdf]

Description of Additional Supplementary Files:

Supplementary Data 1. Functional atomic coordinates for the unit cell of TAPA-TFP COFs (AA-stacking mode).

Supplementary Data 2. Functional atomic coordinates for the unit cell of NH_2-CuBDC MOFs.

Supplementary Movie 1. TAPA-TFP-0.25-NH_2-CuBDC CMOF membrane (playback speed × 2).
